# Inhalable point-of-care urinary diagnostic platform

**DOI:** 10.1126/sciadv.adj9591

**Published:** 2024-01-05

**Authors:** Qian Zhong, Edward K. W. Tan, Carmen Martin-Alonso, Tiziana Parisi, Liangliang Hao, Jesse D. Kirkpatrick, Tarek Fadel, Heather E. Fleming, Tyler Jacks, Sangeeta N. Bhatia

**Affiliations:** ^1^ Koch Institute of Integrative Cancer Research, Massachusetts Institute of Technology, Cambridge, MA 02139, USA.; ^2^ Marble Center of Cancer Nanomedicine, Massachusetts Institute of Technology, Cambridge, MA 02139, USA.; ^3^ Harvard-MIT Division Health Sciences and Technology, Cambridge, MA 02139, USA.; ^4^ Institute of Medical Engineering and Science, Massachusetts Institute of Technology, Cambridge, MA 02139, USA.; ^5^ Department of Biology, Massachusetts Institute of Technology, Cambridge, MA 02139, USA.; ^6^ Howard Hughes Medical Institute, Massachusetts Institute of Technology, Cambridge, MA 02139, USA.

## Abstract

Although low-dose computed tomography screening improves lung cancer survival in at-risk groups, inequality remains in lung cancer diagnosis due to limited access to and high costs of medical imaging infrastructure. We designed a needleless and imaging-free platform, termed PATROL (point-of-care aerosolizable nanosensors with tumor-responsive oligonucleotide barcodes), to reduce resource disparities for early detection of lung cancer. PATROL formulates a set of DNA-barcoded, activity-based nanosensors (ABNs) into an inhalable format. Lung cancer–associated proteases selectively cleave the ABNs, releasing synthetic DNA reporters that are eventually excreted via the urine. The urinary signatures of barcoded nanosensors are quantified within 20 min at room temperature using a multiplexable paper-based lateral flow assay. PATROL detects early-stage tumors in an autochthonous lung adenocarcinoma mouse model with high sensitivity and specificity. Tailoring the library of ABNs may enable not only the modular PATROL platform to lower the resource threshold for lung cancer early detection tools but also the rapid detection of chronic pulmonary disorders and infections.

## Teaser

A portable inhalable diagnostic spots lung cancer in rapid urine test, easing resource inequity in early detection.

## INTRODUCTION

Lung cancer death has continued to decline in nations with a high development index (HDI), due in part to substantial progress in early detection tools and timely therapeutic interception (*1*). However, disproportionately high mortality is observed in lung cancer cases in low- and middle-income countries (LMICs) and correlates with late-stage disease detection, illustrating inequity in early diagnosis in resource-poor settings—one of the chief challenges in addressing cancer health disparities (*2*). Where available, low-dose computed tomography (LDCT) has been the standard of care to screen for early-stage lung cancer among high-risk and asymptomatic people, and this practice has led to an approximately 20 to 25% reduction of mortality in clinical trials (*3*–*5*). Yet, reduced patient access to imaging and the scarcity of trained personnel remain notable clinical challenges in areas outside urban imaging infrastructures in HDI countries, not to mention in LMICs.

Liquid biopsies (LBs) that detect the presence of endogenous, cancer-implicated biomarkers in body fluids, such as shed proteins, circulating tumor DNA (ctDNA), and tumor cells, are emerging as transformative, noninvasive tools to diagnose and monitor lung cancer (*6*–*9*). These assays often rely on resource-intensive analytical technologies such as chromatography, tandem mass spectrometry, next-generation sequencing, and flow cytometry to identify molecular or genetic changes present in captured samples, thus limiting their applicability in low-resource settings. Furthermore, the detection of cancer-associated biomarkers is conditional upon their release into the bloodstream, which places constraints on other activity-based hallmarks of diseases from being used for diagnostic purposes. For instance, proteases are found to play direct functional roles during tumor progression, and display distinct expression and activity signatures across hallmarks of cancer, but are physically located in the tumor microenvironment (TME), and thus might be overlooked by LBs (*10*). We and others have developed exogenous biomarkers—activity-based diagnostics (ABDs)—that leverage catalytic proteases to continuously liberate synthetic barcodes. These catalytically amplified and measurable exogenous reporters reveal the signature of proteolytic dysregulation in the TME that are linked to cancer states and therapeutic responses via imaging, urine biopsy, or breath biopsy (*11*–*18*), thereby expanding the panel of available analytes for more predictive diagnostics. We envision that the development of point-of-care (POC) tests that offer accurate real-time ABD readouts could further unleash their translational potential and alleviate surging demands for early lung cancer detection tools and improve cost-effectiveness of this screening practice, especially in resource-poor settings. Lateral flow assays (LFAs), a well-established platform for POC testing, have been used to help assess at-risk groups by detecting the presence of cancer-associated proteins and genetic mutations in LBs (*19*, *20*). However, cancer-detecting LFAs have been minimally implemented, in part due to the need for multiplexing capacity, the multistep assay protocols necessary to derive quantitative measurements, and the lack of informative biomarkers. Inspired by the multiplexed nature and potential for immobilization of nucleic acids on paper strips, the rational design of chemically stabilized oligonucleotides for molecular barcoding might enable accurate LFA-based readouts of dysregulated proteolysis through fluid biopsy.

In addition to reducing the resource demands of detection assays, diagnostic platforms can also be improved by establishing self-administered formulations, as this would further lower the threshold for clinical deployment. For example, inhaled medicines have been routine tools for at-home treatment of chronic lung diseases for decades (*21*, *22*). This foundation has been leveraged during the design of cutting-edge inhalation technologies [e.g., nebulization and dry powder inhalers (DPIs)] to achieve pulmonary delivery of diagnostics, allowing them to bypass nonspecific systemic degradation and profile early lesions in a proximal manner (*15*, *23*, *24*). Aerodynamic size often dictates the deposition of aerosols in human respiratory tracts and lungs. Larger particles are intercepted by adsorption in the mouth, throat, and upper airways, whereas smaller particles (i.e., 1 to 5 μm) are more likely to deposit in the peripheral lung (bronchi, bronchioles, or alveoli) where lung cancer is predominantly located. Particles <0.5 μm are typically exhaled, and as such, deep-lung deposition of these very small particles is reduced (*25*). Therefore, the complexity of nanoparticle delivery via inhalation arises from the need to quickly convert nanoscale formulations to aerosol clouds with (aerodynamic) size neither too small nor too big—ideally 1 to 3 μm in diameter.

**Fig. 1.**
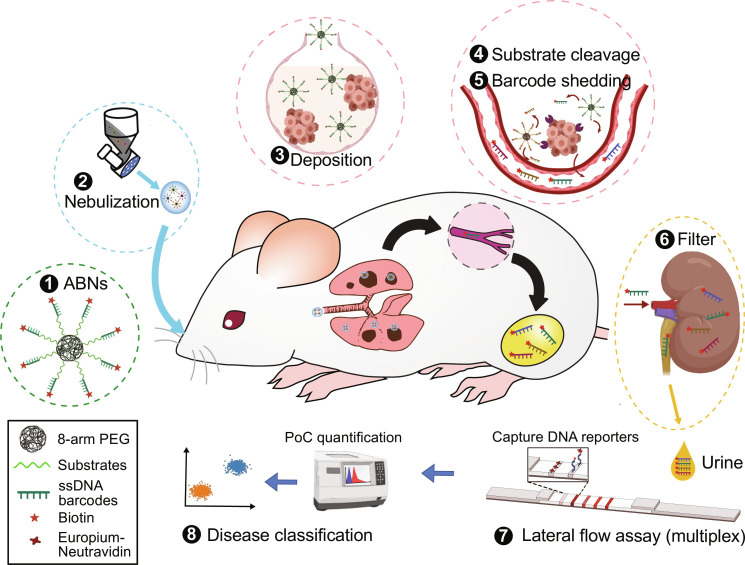
PATROL for early detection of lung adenocarcinoma at the point of care (POC). (1) Single-stranded, phosphorothioate-modified DNA (ssDNA) 20-mers and protease substrates were conjugated to eight-arm PEG nanoscaffolds via click chemistry, creating a cohort of multiplexed ABNs with synthetic DNA barcodes. The DNA barcodes were biotinylated, which can later bind europium (Eu)–Neutravidin complex on paper strips of an LFA. (2) Noninvasive pulmonary delivery of DNA-barcoded ABNs via inhalation. Atomization of nanoscale ABNs into micro-sized aerosols (nano-in-micro process) using a vibrating mesh nebulizer enabled efficient respiratory deposition of inhaled ABNs. The resulting aerosols were introduced into a nose-only inhalation tower with airflow. (3) The aerosols were spontaneously inhaled by mice with early-stage autochthonous lung adenocarcinoma *(**28**)* and deposited on their airways and lung periphery where lung adenocarcinoma nodules reside. (4) The DNA barcodes were quickly liberated upon substrate cleavage by dysregulated proteases associated with early lung adenocarcinoma, and (5) were shed into the systemic circulation. (6) These distinct DNA barcodes in circulation become concentrated via the kidney into the urine. (7) The urine was sampled 120 min after ABN inhalation, and a customized paper-based LFA with multiplexing capacity quantified DNA barcode concentrations present in urine samples at room temperature in a POC manner. The LFA is based on the sequence-specific hybridization at room temperature and labeling of designated DNA barcodes at each of the test lines. The fluorescence of each signal line present on the LFA was quantified using a POC reader, and (8) disease classification was carried out based on principal components analysis (PCA).

Here, we introduce a highly modular platform for early detection of lung cancer in a POC format. PATROL (point-of-care aerosolizable nanosensors with tumor-responsive oligonucleotide barcodes) integrates three modules—low-plex activity-based nanosensors (ABNs), a portable inhalation unit, and a multiplexable paper-based LFA (Fig. 1). To enable precision diagnostics, we combined transcriptomic data from human samples with insights from an earlier, intratracheal diagnostic study in mice (*23*) and nominated protease substrates specific to stage I lung adenocarcinoma to probe for tumor-associated proteolytic signatures. We reengineered DNA-barcoded ABNs into micrometer-sized aerosol formulations to optimize the potential for deposition in human lungs. The inhalable ABN format exhibits excellent aerodynamic performance and offers considerable promise for noninvasive, self-administered human delivery by using clinical nebulizers or handheld inhalers. In a genetically engineered mouse model of lung adenocarcinoma, after individual mice breathed in the nebulized aerosols, ABNs were delivered uniformly to lungs, where the substrates exposed to the TME were selectively cleaved to shed synthetic DNA barcodes into the systemic circulation. The nucleic acid reporters encoding unique tumor proteolytic signatures were subsequently concentrated in the urine via the kidney. For rapid detection of the DNA barcodes at the POC, we engineered LFAs to quantify the multiplexed urinary reporters on a single strip at room temperature, and achieved diagnostic results within 20 min. Through the integration of different technological components, we have established a noninvasive “inhale and detect” approach to accurately diagnose early-stage lung cancer that would not require trained medical personnel, a long duration treatment, or centralized diagnostic laboratories. Furthermore, the high modularity of PATROL enables its potential to be extended to achieve rapid detection of chronic pulmonary disorders and infections.

## RESULTS

### Engineering ABN formulations for inhalable diagnostics

**Fig. 2.**
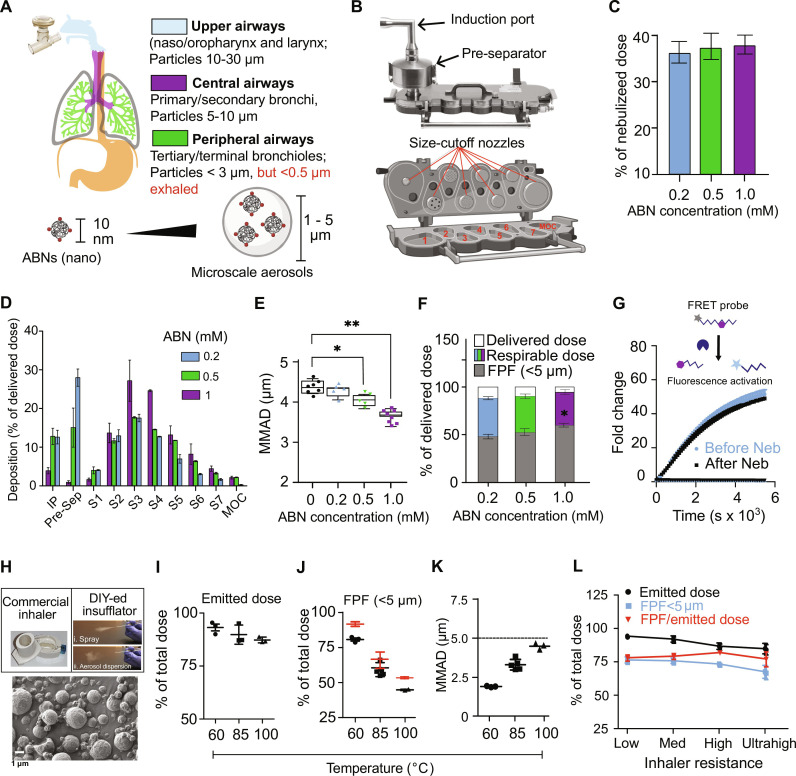
Engineering inhalable ABNs to noninvasively probe protease dysregulation. (**A**) Human adult airways and lung geometry, with predicted aerosol deposition with respect to particle size. (**B**) Next-generation impactor (NGI) and a transverse section, or inside view (below). The NGI classifies aerosols into known size range by drawing aerosols through a cascade of progressively finer nozzles (pointed to by red lines). The air jets from these nozzles affect plane sampling surfaces of stages 1 to 7 (S1 to S7) and the micro-orifice (MOC), and each stage collects finer aerosols than its predecessor. (**C**) The delivered doses were minimally changed with the concentration of ABNs in bulk solution that is represented by peptide substrate concentration = 0.2, 0.5, and 1 mM. (**D**) Aerosol deposition (by mass) of the nebulized ABNs on the NGI. IP, induction port; Pre-Sep, preseparator. (**E**) The median mass aerodynamic diameter (MMAD) of ABN aerosols decreased, but (**F**) the fine particle fractions (FPFs; gray) of nebulized aerosols increased, as the ABN concentration increased. *P < 0.05 and **P < 0.01 by one-way analysis of variance (ANOVA) test with Dunnett’s multiple comparisons to 0 mM in (E) and 0.2 mM in (F). (**G**) The relative release of a quenched fluorescent reporter after incubation with recombinant matrix metalloproteinase 13 (MMP13) was measured for ABN before and after nebulization. Fluorescence resonance energy transfer (FRET)–paired peptides turn on fluorescence when MMP13 cleaves the substrate. (**H**) Scanning electron micrograph of spray-dried, ABN-laden microparticles. The dry powder microparticles can be aerosolized by both commercially available inhalers (top left) and a laboratory-made insufflator (top right). (**I**) Emitted doses, (**J**) FPFs, and (**K**) MMADs of ABN-containing microparticles spray-dried at 60°, 85°, and 100°C. Red dots in (J) were recalculated by dividing FPFs by emitted doses. (**L**) Emitted doses and FPFs are largely independent of inhaler intrinsic resistance.

As particles with 1 to 5 μm in aerodynamic diameter tend to deposit in human peripheral lungs (Fig. 2A) (*26*), we first sought to reengineer ABNs into submicrometer aerosols so as to be suitable for delivery via inhalation (Fig. 2B) via nebulizers. We began by synthesizing a conventional ABN by functionalizing an eight-arm polyethylene glycol (PEG) nanoscaffold with tandem peptides containing a matrix metalloproteinase (MMP) substrate (i.e., LQ81 in table S1) and a Cy7-tagged glu-fibrinopeptide B (GluFib) reporter. Each PEG scaffold carried approximately eight tandem peptides, and the hydrodynamic size ranged from 10 to 15 nm in diameter. We chose nebulizers as a candidate proof-of-concept format, as they require minimal formulation design to generate aerosols, and inhalers were further benchmarked against nebulizers. The model ABNs were dissolved in 0.9% NaCl solution at a range of concentrations up to 2 mM substrate equivalent (or 250 μM PEG equivalent) and then aerosolized with a vibrating mesh nebulizer. We observed that aerosol production by the nebulizer was considerably reduced when the substrate concentration exceeded 1 mM (or 0.125 mM PEG equivalent) and so proceeded with ABN formulations incorporating no more than 1 mM substrates. Nebulizers create aerosols continuously, while aerosols are breathed in only during inspiratory phases. We next sought to quantify the inhaled proportions of ABN aerosols, defined as delivered doses. To measure delivered doses in vitro, a simplified breath simulator was used to create representative profiles (i.e., sinusoidal) of human respiration and the aerosols were collected during inhaling cycles in a next-generation impactor (NGI) (Fig. 2B and fig. S1A)—a simplified model of human tracheobronchial tree and alveoli. The delivered doses of aerosols generated from various ABN concentrations remained largely unchanged at 35 to 38% (Fig. 2C), and the residual volume of condensed aerosols in the nebulizer and T tubing was similar and minimal. An NGI collects aerosol samples in stages as they pass through a cascade of progressively finer nozzles (Fig. 2, B and D), and the mass of aerosols deposited in each stage can be used to estimate aerodynamic size and fine particle dose (Fig. 2D). The median mass aerodynamic diameters (MMADs) of ABN aerosols generated by the vibrating mesh nebulizer were 3.72 to 4.35 μm (Fig. 2, D and E), and the size change was inversely proportional to the ABN concentration. Conversely, the fine particle fractions (FPFs) of these aerosols, a parameter that estimates aerosol fractions that favor peripheral lung depositions, were proportional to the ABN concentrations, culminating at 60.2% of the delivered dose (Fig. 2F). Both delivered doses and FPFs fall within the range of inhalation drugs tested with the same nebulizer. The size and proteolytic sensitivity of the ABNs before and after nebulization were largely unaltered in comparison with the original nanosensors (fig. S1B and Fig. 2G).

To enable high dose delivery to deep lungs and improve cost effectiveness and ease to use of inhalation devices for POC detection, we also explore the feasibility of formulating the nanosensors into portable, electric power–free, and handheld devices—DPIs that are capable of delivering large doses of submicrometer powders (dry aerosols) to the lungs within seconds upon a single breath actuation. We used a particle engineering technology, termed excipient enhanced growth, to make carrier-free and micrometer-sized dry particles (1 to 3 μm) that increase in size following inhalation to minimize upper respiratory tract deposition and maximize targeted deposition in the lung periphery (*27*). A solution of nanosensor cocktails, mannitol (a hygroscopic excipient), and l-leucine (a surface-active dispersion enhancer) was co-sprayed via a spray dryer (fig. S1C), where the solution is heated at the inlet and sprayed through fine nozzles into a dryer, followed by exit from the outlet in the form of submicrometer powders. The resulting microparticles show relatively coarse surface morphology (Fig. 2H). These dry particles can release the nanosensors immediately after reconstitution in simulated lung fluids (fig. S1D), indicating the potential for fast dissolution of the inhaled microparticles when in contact with humid respiratory tract. We then loaded these microparticles into a capsule and inserted it in a clinically used inhaler—RS01. A short period of air flow (approximately 3 to 4 s and contingent on inhaler resistance) mimicking a deep inhalation disaggregates and introduces microparticles into an NGI to assess aerodynamic performance. In the preliminary optimization, we found that inlet temperature (heating the incoming ABN/d-mannitol/l-leucine solution) dictates aerodynamic performance of the inhalable microparticles in comparison with other parameters such as feed flow rate, outlet temperature, and air flow rate. The spray drying at inlet temperature = 60°C yields the highest emitted dose (92.3 ± 3.0% of total mass of microparticles loaded in the capsule; Fig. 2I) and FPF (91% ± 1.3% of emitted dose; Fig. 2J) and the lowest mean mass aerodynamic diameter (2.1 ± 0.1 μm; Fig. 2K). This suggests that nearly all microparticles not only are respirable but also can potentially reach the deep lungs (i.e., regions rich in bronchioles and alveoli). We then assessed the aerodynamic performance of ABN-laden microparticles in DPIs with different intrinsic resistance (low, medium, high, and ultrahigh). Despite that emitted doses and FPFs decrease slightly as inhaler resistance (Fig. 2L), all the inhalers can discharge more than 80% microparticles (emitted dose) and generated 78 to 91% fine particles (of emitted dose) conducive to peripheral lung deposition. Although the low-resistance inhaler seems the best performer in the assessment, high inspiratory airflow rates and respiratory efforts required by such inhalers cannot always be achieved by high-risk or elderly populations suffering chronic pulmonary disorders. The aerosol performance is largely independent of inhalers, indicating highly comparable delivery efficiency and dose uniformity of the inhalable, ABN-containing microparticles across different inhalers. Collectively, these protease nanosensors can be formulated for efficient lung delivery via aerosol through direct nebulization from aqueous solutions, or incorporation into respirable dry microparticles.

### Nominating a panel of ABNs with sufficient power and POC adaptability

**Fig. 3.**
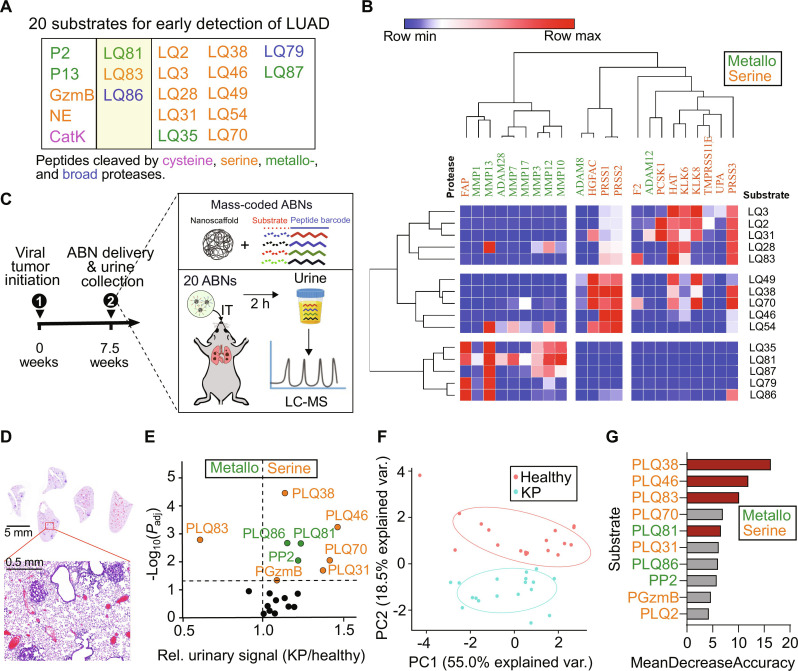
Nomination and down-selection of protease substrates specific to early-stage lung cancer for POC adaptability. (**A**) A panel of 20 peptides (table S1) susceptible to proteolytic cleavage by dysregulated proteases in stage I lung adenocarcinoma. The peptide substrates in two left-most columns have been previously validated as prominent probes in the classification of lung adenocarcinoma in mouse models *(**23**)* and characterizing proteolytic activity of infiltrated myeloid cells, whereas the substrates tabulated in columns 2 to 5 were exclusively nominated for stage I human lung adenocarcinoma. Three peptides (second yellow column) were included in both groups. (**B**) Heatmap of *z*-scored fluorescent fold changes at 50 min (average of two replicates) showing hierarchical clustering of proteases dysregulated in stage I lung adenocarcinoma (horizontal) by their substrate specificities and of selected FRET-paired synthetic substrates (vertical) by their protease specificities. Green and orange denote metallo- and serine proteases, respectively. (**C**) Mass-coded ABNs were delivered into KP mice via intratracheal instillation 7.5 weeks after tumor induction (KP_7.5wk_). The urine samples were collected at 120 min after administration, and the mass-coded reporters were quantified with liquid chromatography–tandem mass spectrometry (LC-MS/MS). (**D**) Hematoxylin and eosin staining shows that lung nodules of KP_7.5wk_ were mainly grade I/II tumors and nodule size <0.5 mm. (**E**) In vivo screen revealed that nine reporters were differentially enriched in the urine of KP_7.5wk_ mice (green and orange dots). (**F**) PCA enabled the successful classification of early lung adenocarcinoma from the healthy cohort using 20-plex ABNs. Each dot represents one mouse in the animal cohort. (**G**) Variable importance analysis ranked the importance of the probes for tumor classification. Probes with higher mean decrease accuracy (i.e., importance scores) produced by the random forest model, contributing more to the diagnostic performance. PLQ38, PLQ46, PLQ83, and PLQ81 (red) are among the most robust probes.

To date, heterogeneous malignancies are typically classified via high-throughput screening of large panels of biomarkers, yet this process requires resource-intensive techniques. However, the design of a POC diagnostic tool requires both facile administration and a low-infrastructure readout. We sought to identify a minimal set of precision probes that could offer high predictive power via simply viable screening operations suitable for decentralized settings, and thereby help inform early detection and initiate timely interception. By leveraging tumor proteases, we established a library of ABNs that can detect early-stage lung cancer by building on our previously validated probes and nominating additional proteases and substrates specific to early lesions (*23*), with suitability for low-plex (up to four) POC assay requirements as our priority. We used DESeq2 to identify differentially expressed proteases in tumors and nontumor adjacent tissues from patients with stage I lung adenocarcinoma (fig. S2A) in The Cancer Genome Atlas (TCGA) database. We found significant protease dysregulation even at the earliest stages of disease, with the top 22 up-regulated proteases listed in fig. S2A. We then constructed a library of 73 peptide substrates mined from the literature and paired them with quenched fluorescent labels to form activatable probes for in vitro screening against the selected, dysregulated proteases (fig. S2B). The probes were incubated with recombinant proteases, and we measured cleavage kinetics as reflected by fold changes in fluorescence signals (illustrated in fig. S2, B and C). Among the 73 nominated substrates, we selected 15 substrates for their selectivity and sensitivity to target proteases (Fig. 3, A and B). We further selected another five peptide sequences from our existing substrate library to augment the capacity to probe the activity of metalloproteases and proteases secreted by infiltrating immune cells (*15*, *16*, *23*). From the in vitro screening, the substrates show high specificity against the family of proteases and preferential selectivity against proteases of the same subtype (Fig. 3B and fig. S2B).

The final expanded panel of 20 candidates was then evaluated via in vivo screening in mouse models in an effort to nominate a small bespoke probe set with low-plex compatibility (Fig. 3A and table S1). Specifically, the set of 20-plex ABNs was synthesized by conjugating mass-coded tandem peptides (GluFib isotope+ substrate peptide) to the PEG nanoscaffolds (table S2). We then evaluated the ABNs in an autochthonous lung adenocarcinoma mouse model that was induced in *Kras^LSL-G12D;^Trp53^fl/fl^* (KP) mice by adenovirus expressing Cre recombinase under the control of the surfactant protein C promoter (*28*, *29*). Previous work established this model as closely recapitulating human disease progression from alveolar adenomatous hyperplasia to grade IV adenocarcinoma (*23*, *28*). The lung tumors were allowed to grow for 7.5 weeks (KP_7.5wk_), at which point lung nodules were histologically categorized as grade I/II and the nodule sizes were typically smaller than 0.5 mm in diameter (Fig. 3D). The pooled 20 ABNs were intratracheally instilled to the KP_7.5wk_ and age/gender-matched healthy mice, and the urine produced 60 to 120 min after ABN administration was collected. The mass-encoded reporters present in the urine samples were analyzed using liquid chromatography–tandem mass spectrometry (LC-MS/MS). As shown in the volcano plot, 9 of 20 probes offer significant detection power (Fig. 3E). Unsupervised dimensionality reduction by principal components analysis (PCA), an algorithm that transforms a large set of variables into a smaller one without losing any important information, also showed that this 20-plex ABN was able to cluster tumor-bearing mice at an early time point (Fig. 3F). Using variable importance analysis with a random forest model, we further identified the top 5 probes (PLQ38 > PLQ46 > PLQ83 > PLQ70 > PLQ81) among those with the greatest fold change (KP/healthy mice) and significance (Fig. 3, E and G). We selected PLQ38, PLQ46, PLQ83, and PLQ81 as an unbiased 4-plex panel. Notably, we excluded PLQ70, although it ranks higher than PLQ81 in the importance list. This choice was made because PLQ38, PLQ46, and PLQ83 are also sensitive to serine proteases (e.g., PRSS, HGFAC, or F2), which cleave PLQ70, whereas PLQ81 is a metalloprotease substrate. We thus selected this subset of four ABNs to reformulate into a low-plex, inhalable panel and assess its diagnostic performance in vivo to detect early-stage lung adenocarcinoma.

### Differentiating early lung adenocarcinoma in autochthonous mouse models with nebulized ABNs

**Fig. 4.**
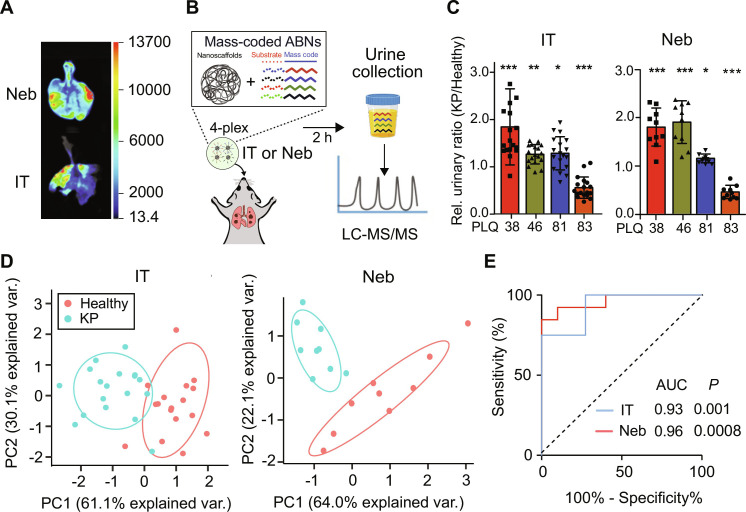
Inhalable ABNs enabled classification of early lung adenocarcinoma. (**A**) Lung local biodistribution of 1 nmol eight-arm PEG nanoscaffolds delivered to wild-type C57BL/6 via nebulization (Neb; top) or intratracheal instillation (IT; bottom), as characterized by fluorescence imaging. The PEG scaffold was labeled with near-infrared dye, Cy7. (**B**) Mass-coded ABNs were delivered into KP mice via either intratracheal instillation or nebulization 7.5 weeks after tumor induction. The urine samples were collected at 120 min after administration, and the mass-coded reporters were quantified with LC-MS/MS. (**C**) Urinary output of 4-plexed mass-coded ABNs intratracheally delivered (KP: *n* = 17; healthy control: *n* = 18) or nebulized (KP: *n* = 9; healthy control: *n* = 9) to KP (KP_7.5wk_) and control mice at 7.5 weeks after tumor induction. *P < 0.05, **P < 0.01, and ***P < 0.001 by Student’s *t* test. Error bars represent SDs. (**D**) PCA of mean-normalized urinary mass reporters for KP_7.5wk_ and control mice treated either with intratracheally delivered ABNs or with nebulized aerosols. Each dot represents a mouse in the animal cohort. (**E**) Receiver operating curve (ROC) curves showing a predictive power of 4-plex urinary reporters from a subset of KP_7.5wk_ and healthy controls in discriminating an independent test cohort of KP lung tumor from healthy controls. A baseline area under the curve (AUC) value of 0.5 represents a random classifier (black dotted line), and a perfect AUC is 1.0. *P* value represents the significance level between the AUC of KP ROC and that of baseline ROC.

We next sought to validate the inhalable ABN formulations in vivo for lung cancer detection. While DPI-based microparticles demonstrate superior potential for deep lung deposition in humans, nebulizers remain a primary candidate for in vivo validation given the lack of DPIs designed for rodents (DPIs are breath-actuated), and thus, the DPI advantage regarding lung deposition patterns cannot be recapitulated in rodent models. As illustrated in fig. S3 (A and B), mice were placed in restrainers of an inhalation tower and they were exposed to nebulized nanosensors only via nasal openings. We first maximized the delivery efficiency of the aerosols by varying flow rates of air influx into the tower, which, in combination with nebulization rates, determined delivered doses. Micropumps that drew air at 25 ml/min mimicked mouse inhaling at three randomly selected ports, and Cy7-tagged model ABNs (i.e., PEG-LQ81-GluFib) were delivered via aerosols (*30*). We found that in comparison with 1, 4, 8, and 20 liters/min, the flow rate at 2 liters/min deposited the highest portion of aerosols to each nasal opening, which was approximately 0.6% of the total dose entering the tower (fig. S4A) over 10 min of nebulization. Higher flow rates led to more accumulated aerosols in the exhaust filter, while lower flow rates (e.g., 1 liter/min) increased residual volume of ABN solutions in the nebulizer and T-tubing hose. We further observed that the nebulized ABNs resulted in a more even distribution throughout the lung periphery, while intratracheally instilled ABNs tended to accumulate in bronchi and central lungs, with high variability across mice in the tested cohort (Fig. 4A and fig. S4, B and C). This high heterogeneity of distribution may overwhelm specific respiratory zones but disregard other areas of the tract, leading to the possibility of missing tumor nodules. We also found that the concentration of released reporters in plasma and urine was higher in the nebulization group (fig. S4, D and E) than that from the intratracheal group when dosed with the same amount of ABNs. Differing from intratracheal instillation, inhaled ABNs that deposit on upper airways were cleared into the gastrointestinal system (fig. S5, A and B) due to mucus entrapment and mucociliary escalation clearance (*13*). To assess whether these ABNs reenter systemic circulation, we orally gavaged the mice with the ABNs and examined pharmacokinetics, biodistribution, and urinary readouts. We found that no fluorescent signals from either cleaved reporters or intact ABNs were detected outside of the digestive organs (fig. S5, C and D), suggesting that the swallowed nanosensors would not yield background signals in the urine that lower diagnostic power. The ABNs in the stomach and intestines were excreted within 24 hours (fig. S5E).

We next examined whether nebulized 4-plex nanosensors can detect the lung adenocarcinoma at early stages and are used as an alternative to intratracheal instillation. The mass-coded 4-plex ABNs were administered via a nebulizer to the KP_7.5wk_ and healthy mice (Fig. 4B). Urinary signals of all four reporters were significantly different when administered via nebulizer (PLQ38 and PLQ83: *P*_adj_ < 0.001; PLQ46: *P*_adj_ < 0.01; and PLQ81: *P*_adj_ < 0.05), indicating significant alterations of proteolytic cleavage signature between KP_7.5wk_ and healthy mice (Fig. 4C). In comparing the diagnostic capacity of the same 4-plex of sensors administered by the two different routes, we observed that unsupervised dimensionality reduction by PCA was able to classify most of the KP_7.5wk_ mice from healthy counterparts in the group of intratracheal instillation and all KP_7.5wk_ mice in the nebulization group (Fig. 4D). We also performed receiver operating characteristic (ROC) analysis such that the area under the ROC curve (AUC) is calculated as measure of classification accuracy, where a perfect diagnostic has an AUC of 1 and a random diagnostic has an AUC of 0.5. ROC analysis also showed that nebulized nanosensors (AUC_neb_ = 0.961) performed statistically equivalent to the intratracheally instilled down-selected (AUC_IT_ = 0.932) library (Fig. 4E). With 100% specificity, the nebulized 4-plex ABNs exhibited sensitivity of 84.6%, slightly greater than 75.5% from the intratracheal instillation group but without statistical difference. In summary, inhalable low-level multiplexing of rationally selected ABNs demonstrates robust power for the early detection of mouse autochthonous lung adenocarcinoma.

### Engineering LFA to enable room temperature multiplexing of synthetic DNA barcodes

**Fig. 5.**
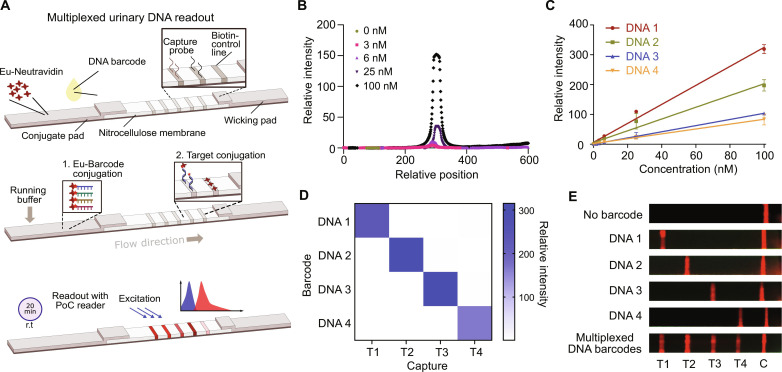
Lateral flow assay (LFA) for the direct multiplexed detection of urinary DNA barcodes at room temperature. (**A**) (Top) Orientation of the LFA, showing the conjugation pad, the nitrocellulose membrane, and the wicking pad. To operate the LFA, urine samples are first placed onto the conjugate pad at least a 5-mm distance away from the spotted Eu-Neutravidin. (Middle) With the application of running buffer, Eu-Neutravidin labels are conjugated with the biotinylated reporter fragments on the conjugate pad and flow laterally to the right, where labeled DNA fragments are captured via sequence-specific hybridization. (Bottom) After 20 min, the LFA strip is inserted into a POC reader, which applies fluorescent excitation and detects the relative intensity of the resulting wavelengths at each designated line location. (**B**) Fluorescence scans across the LFA after the application of varying concentrations of a specific DNA barcode to a strip with a single detection line. The relative intensity was calculated by subtracting the baseline fluorescent signal. (**C**) Operating linear range of four test lines showing immobilized capture probe for each DNA sequence. All barcodes show a linear response from 0 to 100 nM. Relative intensity of each DNA barcode across the concentration range of interest was normalized against its value at 0 nM. (**D**) Selectivity assay of the LFA developed when run against the four different DNA barcodes of 100 nM. (**E**) Representative photographs demonstrating the multiplexing capabilities of the LFA developed (under UV lamps). Individual test strips were all fabricated with four test lines (T1, T2, T3, and T4) and a control (C), biotin line. Each test line represents the capture of distinct probes. Six representative test strips are shown, where the sample administered is indicated to the left of the strip.

To enable the wider deployment of ABN technology in resource-limited settings, we sought to transition to POC detection from the use of peptide-based mass barcodes that require LC-MS/MS for analysis in centralized laboratories. Recently, we reported the use of single-stranded DNA (ssDNA) as barcodes for ABNs (*31*). Here, we sought to design POC assays to quantify the urinary ssDNA barcode signature, with (i) ease of multiplexing, (ii) room temperature operation, and (iii) high sensitivity and selectivity. In Fig. 5A, we outline the design of a multiplexable LFA that operates based on DNA hybridization on a single strip. The target barcodes are 20 nucleotides in length on phosphorothioate backbones with biotin on the 5′ ends. On the LFA, urinary ssDNA barcodes are first tagged with europium (Eu)–labeled Neutravidin, which is preloaded on the conjugate pad. Eu is used as a fluorescent readout for quantitative measurements. The running buffer then carries the Eu-tagged DNA barcodes along the test strip, where the DNA barcodes are subsequently hybridized with their capture sequences, thus forming fluorescing test lines. We also use a positive control line of biotinylated bovine serum albumin (BSA) such that, in the absence of a fluorescent signal at this position, a faulty test result would be inferred. Each test takes about 20 min at room temperature, and the test strips can then be analyzed by a commercial POC fluorescent reader—Axxin AX-2X-S (Axxin, Fairfield, VIC, Australia).

We optimized the physical components of the LFA to improve the sensitivity of the measurement. Specifically, we found that the choice of surfactants used in the running buffer could result in a detrimental effect on the noise level observed on nitrocellulose membranes. As part of the signal optimization process, we screened 24 surfactants against membranes of different pore size and chemical treatment, including commercially available FF80HP, FF80HP Plus, and CN95 (fig. S6A). We first nominated FF80HP Plus with Tween 60 as the surfactant in the running buffer since it resulted in low background fluorescence and minimal conjugate aggregation on the strip (fig. S6, A and B). We then studied various commercially available conjugate pads made of microglass, chopped glass, and polyesters to contain the Eu-labeled Neutravidin. The conjugate pads were laminated onto the nitrocellulose membrane and tested for the release of the Eu conjugates (fig. S7A). Through further optimization, we found that by switching to FF120HP Plus—which has a longer capillary flow time than FF80HP Plus—the fluorescent signal–to–noise ratio of the oligonucleotide barcode was enhanced on the assay. This improvement could be due to the prolonged interaction time between the barcodes and the immobilized capture probes on the membrane (fig. S7B). Figure 5B shows the relative intensity of fluorescent signals on the nitrocellulose membrane where hybridization events occurred with the capture probe, after applying varying concentrations of the corresponding DNA barcode. Notably, the assay was sufficiently sensitive to quantify subnanomolar concentrations of the target. We blocked the Eu-Neutravidin with casein and further adjusted the pH of the running buffer to improve signal performance. We were also able to demonstrate the uniform release of the conjugates into the nitrocellulose membrane with minimum aggregation and background (fig. S7C). Collectively, the LFA optimized for the quantitative multiplexing of ssDNA barcodes operates with FF120HP Plus as the nitrocellulose membrane, STD17 as the conjugate pad, and 0.5% Pluronic F68 in the running buffer at pH 7.4.

The multiplexing capability of the assay was achieved through the spatial printing of individual capture DNA lines, 4 mm apart. We redesigned the dimensions of the LFA (fig. S8A) to be compatible with the commercially available POC fluorescent reader and benchmarked the performance of the reader against that of a benchtop model in the concentration ranges of interest (fig. S8B). We assayed the detection of varying concentrations of the four individual predesigned ssDNA barcodes (i.e., DNA1, DNA2, DNA3, and DNA4 in table S3) when applied to the 4-plex capture strips, and found that the multiplexed LFA had an operating linear range from 0 to 100 nM of DNA1, DNA2, DNA3, and DNA4 (Fig. 5C). It is noted that the sensitivity of the assay for each barcode varies within the linear range, which could be due to (i) the hybridization efficiencies of the DNA targets to their respective captures and (ii) the amount of capture probes on each test line. We then tested the selectivity of the LFA. We challenged the assay with solutions containing 100 nM of each individual DNA barcode and analyzed the relative fluorescence using the POC fluorescent reader between each test. As shown in Fig. 5D, the assay demonstrated high selectivity of the target DNA barcodes. Figure 5E shows the representative images of the LFA when tested against urine samples spiked with individual or pooled DNA barcodes captured with a smartphone under an UV lamp. In summary, we demonstrated that the customized LFA is able to simultaneously capture quantifiable amounts of multiplexed DNA barcodes in urine with high sensitivity and specificity.

### Integrating two POC technologies into PATROL for early detection of lung adenocarcinoma

**Fig. 6.**
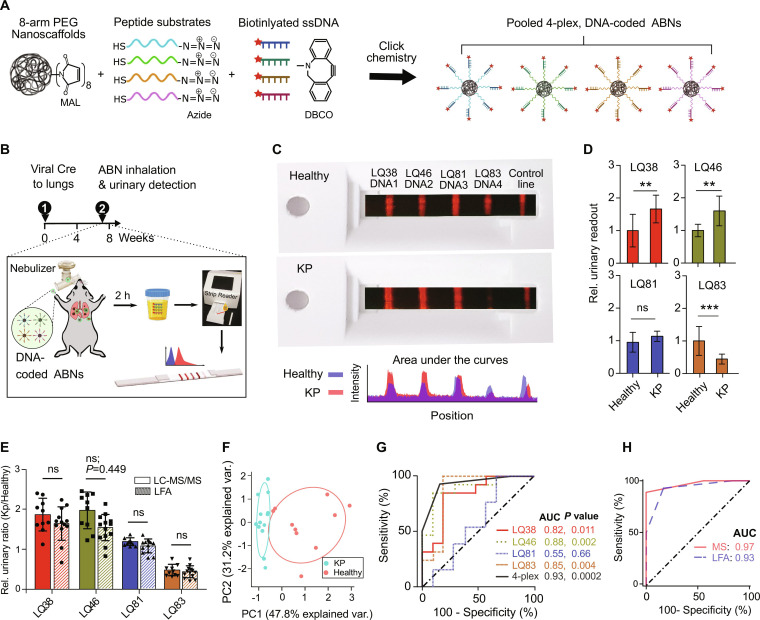
PATROL enabled classification of lung adenocarcinoma at early stage. (**A**) Single-stranded, phosphorothioate-modified DNA 20-mers and selected protease substrates were conjugated to eight-arm PEG nanoscaffolds via click chemistry, creating a cohort of 4-plexed ABNs with synthetic DNA barcodes (i.e., PATROL). (**B**) Nebulization of PATROL into the KP_7.5wk_ lung adenocarcinoma and healthy animals, followed by direct detection of synthetic DNA reporters in urine using the customized LFA. (**C**) Photographs demonstrating the detected urinary DNA reporters from KP_7.5wk_ and healthy mice with the LFA. The images of the LFAs (taken under UV lamp) are overlaid on cassettes (under normal lighting conditions) for the POC reader as a proof-of-concept demonstration. Intensity profile across the representative strips was measured with ImageJ. (**D**) Urinary output of PATROL detected via LFA fluorescence (KP: *n* = 13; healthy control: *n* = 10). *P < 0.05, **P < 0.01, and ***P < 0.001 by Student’s *t* test. Error bars represent SDs. (**E**) Head-to-head comparison of relative ratios of urinary readouts between KP_7.5wk_ and healthy controls (KP/healthy)—signal-to-background ratios. Mass reporters in urine were detected with LC-MS/MS. DNA barcodes in urine were detected with LFA. Statistical analysis was performed between two groups with an unpaired Student’s *t* test. “ns” = not significant. (**F**) PCA of mean-normalized urinary DNA reporters from PATROL for KP_7.5wk_ mice and healthy controls (KP: *n* = 13; healthy control: *n* = 10). (**G**) Receiver operating characteristics (ROC) curves showing a predictive power of a single or 4-plexed urinary reporters in discriminating an independent test cohort of KP lung tumor from healthy controls. *P* value represents the significance level between the area under KP ROC curves (AUC) and that of baseline ROC curves. (**H**) AUC of ROC curves with urinary DNA reporters detected using LFA was comparable to that detected with the LC-MS/MS.

With the individual pieces validated, we sought to integrate the three modules into a single low-resource capable diagnostic platform—PATROL (Figs. 1 and 6A). The DNA-coded ABNs were synthesized by coupling biotinylated DNA barcodes to PEG scaffolds via the peptide substrates (i.e., PLQ38-DNA1, PLQ46-DNA2, PLQ81-DNA3, and PLQ83-DNA4). The ABNs were approximately 15 nm in diameter and were highly negatively charged (refer to fig. S9 for characterizations). Each PEG nanoscaffold was covalently bonded with approximately four tandem peptide-DNA barcodes (fig. S9D). We then continued to use the KP lung adenocarcinoma mouse model (KP_7.5wk_) and attempted to validate urinary DNA reporter detection via the LFA after administering nebulizer-delivered ABNs (Fig. 6B). Figure 6C shows representative photographs of the LFA for a healthy (blue, top) or KP (red, bottom) mouse, as well as the quantification of the fluorescence signals (measured as area under the curve of each peak) for each of the DNA barcodes. With the peak areas, we calculated the concentration of each barcode in the urine using established standard curves (Fig. 5C).

For the cohorts of mice tested, we compared urinary concentration of each barcode between the two groups and plotted the ratios of urinary readouts from KP relative to healthy mice (Fig. 6D). We observed significant differences in the cleavage of PLQ38, PLQ46, and PLQ83 but not in PLQ81 between the healthy and KP mice. Notably, urinary readouts detected with LC-MS/MS and LFA showed no significant difference in signal-to-noise ratios (i.e., KP/healthy in Fig. 6E). Unsupervised clustering was able to classify all the KP_7.5wk_ mice with grade I/II lung adenocarcinoma (Fig. 6F). We again performed the ROC analysis to characterize the predictive power of each probe and their combinations. PLQ38, PLQ46, and PLQ83 showed competent predictive power as a single classifier, based on calculated AUC values of 0.82, 0.88, and 0.85, respectively (Fig. 6G). The overall AUC of the four combined probes increased to 0.93, which was comparable to that of the nebulized 4-plexed ABNs detected via their mass barcodes (AUC = 0.96), although the significance (*P* value) of each probe (except PLQ83) was one order of magnitude lower in the LFA outputs (for DNA) than in the urinary readouts by mass spectrometry (Fig. 6H). With 100% specificity, the sensitivity of the DNA reporters detected by the LFA reached 75.2%, slightly less than that of urinary mass reporters, but remains comparable to the sensitivity (i.e., 75%) of micro-CT that detected lung adenocarcinoma in 7.5-week KP mice at the same specificity (*23*). In summary, diagnostic capacity is retained after switching to PATROL.

**Fig. 7.**
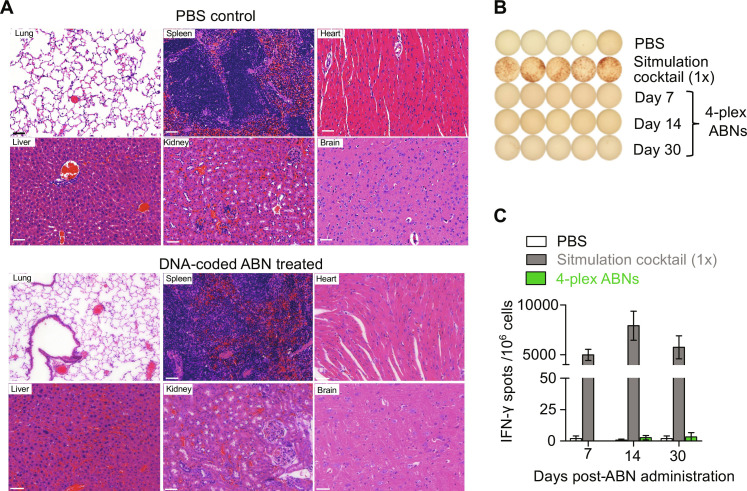
Nebulized nanosensors show no obvious preclinical toxicity or immunogenicity. (**A**) Representative hematoxylin and eosin histological images of excised lung, spleen, heart, liver, kidney, and brain of PBS- and ABN-treated mice. Scale bar, 50 μm. A single dose of 4-plex ABNs, threefold higher than the dose used for detection tests, was nebulized to five healthy mice for toxicity evaluation. The PBS-nebulized mice were used as a control group. (**B**) Representative images of interferon-γ (IFN-γ) ELISPOT assay of PBS-treated, stimulated (positive control), and ABN-treated peripheral white blood cells. The wild-type mice were nebulized with the same dose of ABNs in (A). In the positive control, the white blood cells isolated from healthy and sex- and age-matched C57BL/6J mice were stimulated with eBioscience cell stimulation cocktail (1×). The eBioscience cell stimulation cocktail (500×) contains 40.5 μM phorbol 12-myristate 13-acetate (PMA) and 670 μM ionomycin in ethanol. Days 7, 14, and 30 denote the number of days after ABN administration. (**C**) Quantification of IFN-γ spots in (B) of untreated and treated samples revealed nonimmunogenicity.

As an additional preclinical step, we examined the safety profile of a high dose of inhalable ABNs with DNA barcodes (threefold higher doses than tested above). Neither general toxicity nor clogging of vasculature was observed in major organs of wild-type mice 7 days after a single dose of ABNs delivered via nebulization, as indicated in weight monitoring (fig. S10, A and B) and histological assessment of principal tissues (Fig. 7A). We further evaluated interferon-γ (IFN-γ) secretion, a major inflammatory cytokine produced by activated immune cells, and considered an indicator of immunogenicity of an administered substance. At both shorter (i.e., 7 days) and longer (14 and 30 days) intervals after ABN dosing, peripheral blood mononuclear cells were isolated and an enzyme-linked immunospot (ELISPOT) assay was performed. Upon stimulation with the same panel of the ABNs, we found that no IFN-γ was secreted by circulating lymphocytes at any tested time points, consistent with the interpretation that each module of the inhaled ABNs (i.e., peptide substrates, PEG scaffolds, and DNA barcodes) was nonimmunogenic (Fig. 7, B and C).

## DISCUSSION

Although there has been substantial progress in lung cancer diagnosis and treatment over the past several decades, progress has not kept pace in tackling cancer health disparities, including the low rates of screening in low socioeconomic groups and geographically isolated areas. A strategy to address such inequity is to lower the technological threshold for a patient’s access to screening programs and early detection. This work established PATROL as a POC detection platform that integrates modular inhalable synthetic biomarkers and multiplexable LFAs to detect, in a noninvasive manner with low-infrastructure needs, dysregulated proteolytic activity that correlates with early-stage lung cancer (Figs. 1 and 3, B and E). We first formulated the ABNs into microscale aerosols that could be delivered with clinical nebulizers or inhalers and are conducive to deep lung deposition. We demonstrated the inhaled ABNs maintained robust in vivo diagnostic power in a genetically engineered mouse model of lung cancer. Next, we downsized a large library of stage-tailored ABNs to a 4-plex cohort and reengineered them with synthetic DNA barcodes to enable easy multiplexing with LFAs at room temperature. Finally, we validated the highly modular “inhale and detect” PATROL platform to discriminate grade I/II lung adenocarcinoma in an autochthonous model with high sensitivity and specificity (AUC = 0.93; Fig. 6, F and G). Collectively, PATROL holds great clinical potential not only to attain both sensitive and specific lung cancer detection at early stages but also to enable easy deployment in resource-limited settings.

Activity-based synthetic biomarkers that selectively leverage protease activity in tumors have yielded amplified signals and outperformed blood biomarkers in detecting smaller tumors in several preclinical models, such as carcinoembryonic antigen in colorectal cancer (*32*) and human epididymis protein 4 in ovarian cancer (*33*). In addition to output augmentation, PATROL increased detection specificity of the protease-responsive probes by identifying protease signatures that stratify early-stage lung adenocarcinoma using TCGA transcriptomic data (Fig. 3B and fig. S3C). Finally, in vivo screening of nominated ABNs results in a robust low-plex probe set for POC assay compatibility. We envision that recent advances in precision multi-omic sequencing will shed new insight into unique protease dysregulation and heterogeneity in various stages and subtypes of lung cancer. The delivery of ABNs via inhalation provides a means of maximizing their deposition to the lungs and decreases background signals caused by nonspecific enzymatic activation in circulation. The fully noninvasive inhalation delivery also sets the platform a step closer to clinical translation for direct delivery of diagnostic agents to the lungs. The relatively homogeneous deposition of inhaled ABNs to the respiratory system (Fig. 4A and fig. S4, B and C) may also confer the capacity to profile protease activity of tumor nodules at unknown locations or within the otherwise-inaccessible periphery of the lung. By tailoring aerodynamic size of formulations and rational selection of inhalational devices for subjects, it should be feasible to push the particle deposition down the tracheobronchial tree to alveolar regions, or reverse the deposition pattern to favor the upper airways (Fig. 2, E, F, and J to L).

Conventional LFAs often face limitations in quantitative high-throughput analysis of biomarkers. To overcome this shortcoming, PATROL uses the most relevant protease substrates to maintain the specificity of a low-plex assay and leverages 20-mer ssDNA sequences as barcodes. Dedicated designs of DNA sequences and the optimization of modules into LFA “kits” (fig. S8) enabled us to detect and quantify urinary barcodes at nanomolar levels (Fig. 5, C and D). Sequences of 20 nucleotides offer the capacity to extend the platform to hundreds of orthogonal codes, and thus enable us to quickly ramp up multiplexing capacity when needed (*31*). The synthetic DNA barcodes excreted into the urine are quickly captured on paper strips via direct hybridization with their complementary sequences at room temperature. Hence, the LFA tests offer convenient operation without the need for amplification steps used in polymerase chain reactions and CRISPR-based methods, and can be completed within 20 min in ambient conditions (*34*, *35*). We have also previously described the use of CRISPR-Cas12a for the detection of synthetic ssDNA barcodes (*31*). The main advantage of the technique described here is the multiplexed detection of synthetic ssDNA barcodes in a single LFA strip, which is currently difficult to perform with the CRISPR-Cas approach due to the nonspecific cleavage activity upon target recognition. Furthermore, Cas protein stability and storage could be challenging, especially in low resource settings. We demonstrated that by switching from the CRISPR-Cas12a technology to the direct, room temperature hybridization method with Eu-Neutravidin, the label retains its sensitivity (subnanomolar of DNA; Fig. 5, B and C) and diagnostic specificity (Fig. 6, F to H) when used to screen for lung cancer in our model. Compared to the measurement of mass barcodes by LC-MS/MS, we observed a slight sensitivity decrease in terms of the fold change of some urinary reporters observed between tumor-bearing and healthy mice, such as PLQ81 (Figs. 4C and 6D). Given that the hybridization efficiency of captured DNA varies within a linear range (Fig. 5, B and C), we envision that pairing LQ81 with a more sensitive DNA barcode (e.g., DNA1) may help to amplify the relatively nuanced differences in the activity of some target proteases between tumor and healthy groups. Moreover, the optimization of DNA reporters and capture sequences via iterative processes of sequence design and validation at in vitro, ex vivo, and in vivo levels would further enhance their binding affinities. In contrast to conventional pregnancy tests and coronavirus disease 2019 (COVID-19) rapid antigen tests where a positive or negative readout is a quantum yes/no result, our multiplexed LFAs require the relative quantification of multiple barcodes with which unsupervised algorithmic methods are used to differentiate a cancer diagnosis from healthy groups. Therefore, for future clinical applications, it is essential to define reliable baselines of these urinary DNA barcodes in lung cancer–free groups, often in the context of other benign or noncancerous comorbidities.

While these results highlight the encouraging potential to use PATROL for screening and early detection of lung cancer, future work remains to be accomplished to improve PATROL’s performance. First, the inhaled ABNs mainly profile protease activity at the periphery of tumor nodules. By enhancing the capacity for PATROL to penetrate into deep tumor locations by attaching a tumor-penetrating enhancer (e.g., iRGD peptide), or tuning the size and geometry (e.g., nanorods) of nanoscaffolds (*36*, *37*), we may not only increase the magnitude of detected signals but also enable the sensors to report unique proteolytic signatures present in the tumor cores. In addition, ABNs can be fine-tuned so as to optimize surface presentation of their substrates (e.g., presentation distance to the nanoscaffold surface and/or substrate density per nanoscaffold may modulate enzymatic cleavage kinetics) (*33*) in combination with active targeting to tumor cells and/or microenvironments may further enhance detection sensitivity to monitor for minimal residual disease or early-stage lung cancer. Additional engineering of peptide substrates may further improve the specificity to lung adenocarcinoma. For instance, a tandem substrate with an AND logic gate could enable the liberation of DNA barcodes only upon dual cleavage by two concurrently dysregulated TME proteases. Moreover, the genetically engineered mouse models used in this proof-of-concept report represent only a histological subset of human lung cancer (i.e., adenocarcinoma). Additional efforts must be undertaken to test other subtypes, such as squamous or large cell carcinomas, and to further establish the utility of PATROL for detecting more heterogeneous forms of human lung cancer, also often accompanied by chronic comorbidities. Second, despite the finding that DNA barcodes were cleared from murine lungs within 48 hours of administration via nebulizer (fig. S11A), longitudinal safety studies of the PEG, synthetic peptides, and DNA reporters that make up PATROL nanoparticles should receive comprehensive scrutiny (*23*). Another modification that might help reduce the regulatory threshold is to use endogenous materials to construct the nanoscaffold (e.g., phospholipids). Although the current PATROL LFAs measure synthetic DNA barcodes in the urine with low-level multiplexing on a single strip, further product development may focus on the expansion of multiplexing capacity and consider clinical uses in decentralized settings. For example, the design of custom-made cassettes could take advantage of the advancement of printing and manufacturing capabilities to expand multiplexing capacity on the same device. Adaptors that offer connectivity to smartphones or wearable devices that enable fluorescence readouts may also be developed to allow for even more robust POC use (*38*). Finally, many other confounding factors present in clinical samples, such as pH changes, albuminuria, dietary factors, and patients’ hydration status, may alter testing performance of PATROL. While we believe that such impacts will likely be minimal, as only 5 μl of urine sample is balanced by 125 μl of running buffer, further validation experiments that take these variables into account will be necessary before the clinical implementation of PATROL.

In summary, highly modular diagnostic kits such as PATROL that can offer test results in a single session would be transformative in the detection of lung malignancies. PATROL may also enable efforts to improve risk stratification of early lesions to determine the clinical selection of follow-up procedures. The versatility and modularity of PATROL could also be applied to other pulmonary diseases where proteolytic activities are dysregulated or diagnosis is based on exclusion, such as idiopathic pulmonary arterial hypertension. We envision that by releasing disease screening from its current resource-intensive environment, we may enable feasible surveillance testing that would identify a disease when it is still easy to treat.

## MATERIALS AND METHODS

All chemical reagents were purchased from Millipore-Sigma (Burlington, MA) unless specified.

### Particle size measurement of nebulized nanosensors by dispersion laser diffraction

Volumetric size of particles was measured using a Sympatec HELOS/RODOS (Sympatec GmbH, Clausthal-Zellerfel, Germany) using the rotary feeder and R3 lens (0.9 to 175 μm). Powder was hand-filled into a u-shaped groove of the rotating table to cover a length of approximately 1 cm. The sample passed under a plough scraper and roller to remove any excess and was subsequently drawn up into the dispersing line via the protruding aspiration tube from a static bed. During sample delivery, the rotating table was maintained at a constant rotation setting of 20%. The measurement was set to trigger when the optical concentration (Copt) exceeded 1.1% and cease when the Copt fell below 1% for 5 s (or 60 s real time). The time base was 100 ms, and a forced stability of “4” was applied. The primary pressure was manually set using the adjustment valve in the range 0.2 to 4.5 bar, and three measurements were taken at each pressure setting using freshly loaded powders. Particle size distributions [Dv10, Dv50, Dv90, and volumetric mean diameter (VMD)] were calculated using Fraunhofer theory and analyzed in WINDOX 4.0 software. Particle size measurements for a complete titration curve were made on a single day. Dv10, Dv50, and Dv90 define the size point below which 10%, 50%, and 90% of the material is contained, respectively.

### Particle sizing measurement and fine particle dose of nebulized ABNs using NGI

The aerodynamic size of inhalable ABN formulations was determined with an NGI (Copley Scientific, Nottingham, UK). The preseparator (PS) and plates were precoated with silicone greases to minimize the bounce of particles on their surface. Mass-coded, Cy7-labeled model ABN (PEG_8_40k-LQ81-GluFib) in 0.5 ml of normal saline at different concentrations was loaded into an Aeroneb vibrating mesh nebulizer (Kent Scientific) at different concentrations (PEG_8_40k concentration: 25, 62.5, and 125 μM or peptide substrates = 0.2, 0.5, and 1 mM). Under a standardized breathing pattern of 500-ml tidal volume, 1:1 inhalation:exhalation (I:E) ratio, and 15 breaths per minute frequency, which imitates human breathing cycle—inspiration and expiration, the solution was nebulized into NGI at a flow rate of 15 liters/min (fig. S1A). The nebulizer (bottom part) and T tubing, induction port (IP), PS, eight stages, and nylon filter were carefully rinsed with 3 ml of deionized (DI) H_2_O and then measured for Cy7 fluorescence at excitation/emission = 735/780 nm (note: a necessary dilution may be required for some samples). Delivered dose represents a fraction of total nebulized aerosols that are inhaled by subjects under the standardized breathing pattern. FPF, the fraction of particles with aerodynamic diameter < 5.0 μm implying the fraction of aerosols that tend to deposit on peripheral lung regions (Fig. 2A), was calculated with particle sizing data acquired with an NGI (Fig. 2D), using the following equation:

$$\text{FPF}=\frac{\text{Dose of ABNs with particle size}<5\;\mu m}{\text{Total dose of drug deposited}\;\text{on}\;\text{IP},\text{PS},\text{stage}\;0-7,\text{and}\;\text{MOC}}$$

Mean mass aerodynamic diameter, the aerodynamic particle size of the cumulative percentage of 50% in cumulative particle distribution, was obtained by plotting probability (cumulative percentage of deposited aerosols on stages) against log (upper cutoff diameter of each).

### Spray drying of ABN-laden microparticles

ABNs (4 mg), d-mannitol (12 mg), and l-leucine (4 mg) were dissolved in 5 ml of DI H_2_O and then spray-dried using B-290 Mini Spray Dryer (BUCHI) with the following parameters: inlet temperature = 60°C, outlet temperature = 39° to 44°C, atomizing nitrogen flow rate = 35 mm (473 liters/hour), pump ratio = 10% (3 ml/min), aspiration rate = 70% (100% = 35 m^3^/hour), and nozzle cleaning = 0. The spray-dried microparticles were collected at the end of the gas cyclone. The resulting microparticles were further dried overnight to remove residual H_2_O in a FreeZone benchtop freeze dryer (Labconco) with the cold well set at −80°C and the vacuum degree at 0.02 to 0.015 mbar. The dried samples were then stored in a Corning PYREX top glass desiccator sealed with silicone lubricant, and Drierite was used as a desiccant. ABN-containing microparticles were spray-dried at inlet temperatures 85° and 100°C, with all other parameters kept the same.

To measure the emitted dose, FPFs, and MMADs, 15 mg of microparticles was loaded into a capsule and tested with a monodose RS01 inhaler, which was then connected to the IP of the NGI. To reach 4-kPa pressure drop over the inhaler, the flow rate was adjusted and recorded as FR. The flow controller was then set to run for 240/FR seconds. The DPI, IP, PS, eight stages, and nylon filter were carefully rinsed with 3 ml of DI H_2_O and then measured for Cy7 fluorescence at excitation/emission = 735/780 nm. Emitted dose is a proportion of microparticles discharged from an inhaler with respect to total dose. For DPIs,

$$\text{Emitted dose}=\frac{\text{Dose of ABNs released into}\;\text{NGI}}{\text{Total dose of ABNs loaded into}\;a\;\text{capsule}}$$

and FPFs and MMAD were calculated as mentioned above.

To test the impact of inhaler resistance on the FPFs, the low-, medium-, high-, or ultrahigh-resistance inhalers require approximately air flow rate = 90 liters/min, 68 liters/min, 53 liters/min, or 33 liters/min in the NGI to reach 4-kPa pressure drop over the inhaler, but the exact flow rate must be finely adjusted.

### Nanosensor stability and activity after nebulization

Analysis of nanoparticle stability following nebulization used 0.1 ml of 125 μM Cy7-labeled LQ81 PEG_8_40kDa nanosensors (fig. S2B). The ABN was nebulized into a thoroughly washed glass beaker. The collected nanosensors were measured for hydrodynamic size and zeta potential with a NanoZS Zetasizer (Malvern). Protease cleavage assays of aerosolized nanoparticles used LQ81 PEG_8_40kDa nanosensors with FAM and QSY21 as a quenched fluorescence resonance energy transfer (FRET) pair. The nebulized nanosensors were diluted to 1 μM and incubated with 12.5 nM recombinant human MMP13 (Enzo). Proteolytic cleavage of substrates was quantified by increases in fluorescence over time by a Tecan Infinite M200 Pro fluorimeter. Enzyme cleavage rates were quantified as relative fluorescence increase over time normalized to fluorescence before addition of protease, and the results were benchmarked against untreated nanosensors.

### Oral gavage of ABNs

Cy7-labeled LQ81-GluFib peptide with a cysteine at C terminus was synthesized by CFC Scientific. The peptide was then conjugated to a 40-kDa eight-arm PEG maleimide nanoscaffold (Jenkem Technology) overnight at room temperature as a model ABN. The resulting crude product was then first concentrated to approximately 0.5 ml with an Amicon 0.5-ml, 10-kDa, ultra-centrifugal filter (EMD Millipore), followed by the purification using a Cytiva ÄKTA fast-protein liquid chromatography system equipped with a Sephadex 30 Increase 10/300GL column. The purified model ABN was reconstituted in the phosphate-buffered saline (PBS) (1×, pH 7.4) and diluted to 20 μM (substrate equivalent). Fifty microliters of the model ABN in the PBS was administered with a flexible plastic tubing oral gavage needle from GavageNeedle (Phoenix, AZ). One cohort of mice (*n* = 3) was used to collect blood samples retro-orbitally with heparinized capillary tubes at different time points. The 40-μl (two to three drops) blood from each mouse was mixed with 60 μl of heparin solution (10 U/ml), and the mixture was centrifuged at 1000*g*. Fifty microliters of plasma (including heparin solutions), the liquid portion of blood, was collected and measured for Cy7 fluorescence with a LI-COR Odyssey DLx imager (LI-COR Biosciences). The same dose of ABN was also injected intravenously, and blood was collected as mentioned above for comparison. In parallel, orally gavaged mice were euthanized at 1, 4, and 24 hours, and lung, heart, stomach, small intestine, liver, spleen, kidney, and esophagus were carefully excised for the biodistribution evaluation using a LI-COR Odyssey DLx imager.

### Generation of autochthonous lung adenocarcinoma on mouse models

To induce lung tumors that resemble the human non-small cell lung cancer (NSCLCs) histopathologically, we first generated the *Kras^LSL-G12D^;Trp53^fl/fl^* C57BL/6 (KP) mice in which the activation of an oncogenic allele of *Kras* is sufficient to initiate the tumorigenesis process, and additional deletion or point mutation of *p53* substantially enhances tumor progression, leading to a more rapid development of adenocarcinomas that have features of a more advanced disease. Lung tumors were initiated by intratracheal administration of 50 μl containing adenovirus-SPC-Cre [2.5 × 10^8^ plaque-forming units (PFUs) in Opti-MEM with 10 mM calcium chloride (CaCl_2_)] in female or male KP mice (between 8 and 16 weeks old) under isoflurane anesthesia. Control cohorts consisted of age- and sex-matched mice that also underwent intratracheal administration of AdenoCre. The KP mice were allowed for tumor growth until experiments were performed. All animal studies and procedures were approved by the Committee for Animal Care at Massachusetts Institute of Technology (MIT).

### Gene expression analysis

Human RNA-sequencing data from TCGA Research Network were downloaded from https://www.cancer.gov/tcga. The list of human extracellular protease genes was obtained from UniProt using the following query: (keyword:“Protease [KW- 0645]”) locations:(location:secreted) AND reviewed:yes AND organism:“*Homo sapiens* (Human) [9606]”). Differential expression analysis on the TCGA data was performed using the DESeq2 differential expression library in the R statistical environment. Differentially expressed genes were filtered to have *P*-adjusted value <0.05 and to contain only extracellular proteases and were ranked as a function of log_2_(fold-change stage I LUAD/NAT expression) (Fig. 3B). Proteases were color-coded as a function of protease class.

### In vitro screening of substrate peptides against LUAD-associated proteases at early stages

Fluorogenic protease substrates were synthesized using Millipore-Sigma’s customer peptide library service PepScreen (LQx) or by CPC (PPx, GzmB, NE, CatK) (table S1). Recombinant proteases were purchased from Enzo Life Sciences, R&D Systems, and Haematologic Technologies. For recombinant protease assays, fluorogenic substrates LQx (60 μM final concentration) or PPx, GzmB, NE, and CatK (1 μM final concentration) were incubated in 30-μl final volume in appropriate enzyme buffer, according to manufacturer specifications, with 12.5 nM recombinant enzyme at 37°C (fig. S2B). Proteolytic cleavage of substrates was quantified by increases in fluorescence over time by a fluorimeter (Tecan Infinite M200 Pro). Enzyme cleavage rates were quantified as relative fluorescence increase over time normalized to fluorescence before addition of protease. Hierarchical clustering was performed in GENE-E (https://software.broadinstitute.org/GENE-E/, Broad Institute), using *z*-scored fluorescence fold changes at 60 min.

### Synthesis and characterization of DNA-coded, multiplexed ABNs

All aqueous buffers were deoxygenated by a succession of 30-min vacuum and 15-min dry nitrogen purge. Each of azide-functionalized peptide with a C-terminal cysteine (CPC Scientific, San Jose, CA) was dissolved in dimethylformamide (DMF) to reach 10 mg/ml. Twenty microliters of the peptide solution was then slowly added to 380 μl of phosphate buffer (pH 7.0, 0.2 M), followed by an addition of 500 μl of 200 μM DNA barcode (Integrated DNA Technologies, Coralville, IA). The solution was stirred slowly under the protection of dry nitrogen at room temperature for 1 hour. A 100-μl phosphate buffer containing 0.82 mg of 40-kDa eight-arm PEG maleimide (Jenkem Technology, Beijing, China) was then added dropwise, and the reaction was stirred overnight at room temperature. The resulting crude product was then first concentrated to approximately 0.5 ml with an Amicon 0.5-ml, MWCO (molecular weight cutoff) 10-kDa, ultracentrifugal filter (EMD Millipore, Burlington, MA), followed by the purification using a ÄKTA fast-protein liquid chromatography system equipped with a Sephadex 30 Increase 10/300GL column (Cytiva, Groton, CT). The fractionates eluted from 6.5 to 10 ml were combined and concentrated with an Amicon 0.5-ml ultracentrifugal filter (MWCO 10 kDa). The molar concentration of DNA was determined with the Quant-iT OliGreen ssDNA Reagent kit (Thermo Fisher Scientific, Waltham, MA) according to the manufacturer’s protocol.

Mass-assisted laser desorption/ionization-time of flight mass spectrometry (MALDI-TOF) was performed on a Bruker UltrafleXtreme mass spectrometer (Bruker Corporation, Billerica, MA) to confirm the molecular weights of necessary intermediates and purified products. Sinapinic acid was used as a matrix compound for MALDI-TOF. Hydrodynamic sizes and surface charges of ABNs were measured using a Malvern Zetasizer Nano ZS (Malvern, UK). The geometry and size of ABNs were also visualized with a JEOL2100F cryo-transmission electron microscope (Tokyo, Japan).

### Intratracheal administration of 20 multiplexed ABNs to the KP mice

A library of 20-plexed, mass-coded ABNs by enriching the peptide reporter glutamate-fibrinopeptide B (GluFib) (EGVNDNEEGFFSAR) with different distributions of stable isotopes for in vivo experiments (table S2) were synthesized and characterized by CPC Scientific (Sunnyvale, CA). All in vivo administration of ABN experiments were performed in the morning and were in strict accordance with institutional animal guidelines. Bladders of mice were voided immediately before nanosensor administration. Mice were anesthetized with isoflurane inhalation (Zoetis, Parsippany-Troy Hills, NJ) and were monitored during recovery. For intratracheal instillation studies, a volume of 50 μl of multiplexed (20-plex or 4-plex), mass-coded, or DNA-coded ABN cocktail (20 μM substrate equivalent) in phosphate buffer (0.28 M mannitol, 5 mM sodium phosphate monobasic, 15 mM sodium phosphate dibasic, pH 7.2 to 7.4) was administered by solution injection following intratracheal intubation with a 22-gauge flexible plastic catheter (Exel International, Redondo Beach, CA) and 100 μl of air was then pushed into mouse lung lobes via a 1-ml syringe to ensure a full delivery of ABNs into the lungs. A subcutaneous injection of 200-μl sterile PBS was followed in favor of urine production. Bladders were voided again at 60 min after nanosensor administration, and urines produced from 60 to 120 min after administration were collected using custom tubes in which the animals rested on 96-well plates that collect urines. The urine on every plate was pooled together as a sample from one mouse. All urine samples were immediately frozen at −80°C until analysis by LC-MS/MS or by customized LFA kits.

### Aerosolization of multiplexed ABNs

Exposure of KP and healthy mice to aerosols containing 4-plex, mass-coded, or DNA-coded ABNs was performed in the nose-only inhalation tower system (CH Technologies, Westwood, NJ), including an aerosol distribution chamber and a number of animal containment tubes. Mice were first acclimated to containment tubes by keeping them in the tubes for 15 min and twice a day for three consecutive days before inhalation experiments. Such training mitigates animals’ stresses and ensures normal breathing frequencies and cycles during the restraint. In the morning of experiment day, bladders of the mice were voided of urines and then gently loaded into the tubes that were connected to the ports of the inhalation tower. Four-plex ABN cocktails (125 μM per nanosensor) in 0.5 ml of normal saline were added into an Aeroneb mesh-vibrating nebulizer, and the mice were exposed for 15 to 20 min to aerosols containing ABNs that were generated via the nebulizer and drifted into the tower along with dry airs at a flow rate of 1 liter/min. To determine inhaled dose, an all-glass impinger (AGI; catalog no. 7541-10, Ace Glass, Vineland, NJ) containing 10 ml of PBS + 0.001% antifoam was attached to the chamber and operated at 6 lpm, −6 to −15 psi. Particle size was measured once during each exposure at 5 min using the Aerodynamic Particle Sizer (TSI, Shoreview, MN) operating at 5 lpm. A 5-min air wash followed each aerosol, after which animals were returned to their cage. AGI samples were evaluated to determine the concentration of nanosensors recovered from the aerosol. The inhaled dose was determined as the product of the aerosol concentration, duration of exposure, and the minute volume of individual mouse. Minute volume was determined using Guyton’s formula. A subcutaneous injection of 200 μl of sterile PBS followed aerosol delivery of nanosensors in favor of urine production. Bladders were voided again 60 min after the end of nebulization, and urines produced 60 to 120 min were collected in the custom tubes. The urines were frozen at −80°C until analysis by LC-MS/MS (for mass-coded ABNs) or by customized, paper-based LFA kits (for DNA-coded ABNs).

### Quantification of mass-coded urinary reporters with LC-MS/MS

LC-MS/MS was performed by Syneos Health (Princeton, NJ) using a Sciex 6500 triple quadrupole instrument. Briefly, urine samples were treated with ultraviolet (UV) irradiation to photocleave the 3-amino3-(2-nitro phenyl)propionic acid (ANP) linker and liberate the GluFib reporters from residual peptide fragments. Samples were extracted by solid-phase extraction and analyzed by multiple reaction monitoring by LC-MS/MS to quantify concentration of each GluFib mass variant. Analyte quantities were normalized to a spiked-in internal standard, and concentrations were calculated from a standard curve using peak area ratio (PAR) to the internal standard. Mean normalization was performed on PAR values to account for mouse-to-mouse differences in ABN inhalation efficiency and urine concentration.

### Immobilization of capture probes on the multiplexed LFA

Capture oligonucleotides C1, C2, C3, and C4 (free of amine byproducts) synthesized by Integrated DNA Technologies (Coralville, IA) were reconstituted in 500 μl of 0.1 M borate buffer of pH 9.3 (DCN, Carlsbad, CA). The amine group was activated for BSA (Equitech-Bio, Kerrville, TX) conjugation with 5 ml of *p*-phenylene diisothiocyanate (PDITC) dissolved in dimethyl formamide (DMF) at a concentration of 20 mg/ml. The reaction mixture was kept in the dark at room temperature followed by the addition of 9 ml of *n*-butanol and 15 ml of nuclease-free water. The samples were mixed well and centrifuged at 15,000*g* for 5 min. The yellow layer was discarded, and the extraction process was repeated. The samples were then freeze-dried. To conjugate the activated capture oligonucleotide to BSA, 48.5 nmol of C1, 54.2 nmol of C2, 64 nmol of C3, and 48.5 nmol of C4 were added to 0.20, 0.224, 0.264, and 0.2 ml of 2 mg/ml BSA, respectively. The reaction was done overnight at room temperature in the dark and dialyzed against 10 mM phosphate buffer four times. The conjugates C1, C2, C3, and C4 were diluted 4, 16, 8, and 32 times, respectively, with 10 mM acetate buffer at pH 5.0 (DCN, Carlsbad, CA) and printed on the nitrocellulose test strip (FF120HP Plus) with Biodot (Irvine, CA) programmed to stripe at 0.6 μl/cm at 10 mm/s. The relative position of capture C1, C2, C3, and C4 was 5, 9, 13, and 17 mm, respectively, from the edge of the membrane. Likewise, biotin-BSA (DCN, Carlsbad, CA) of 0.15 mg/ml was also printed on FF120HP Plus (Cytiva, Marlborough, MA) at 21 mm away from the edge of the membrane (control line). The membranes were dried at 40°C for 30 min before placing them into a foil pouch with desiccants (DCN, Carlsbad, CA). It is noted that C1, C2, C3, and C4 are capture probes for DNA1, DNA2, DNA3, and DNA4, respectively.

### Preparation of the Eu-Neutravidin label

The solution in the 1% Eu bead (Thermo Fisher Scientific, Waltham, MA) stock was replaced with 500 μl of 0.1 M 2-(*N*-morpholino)ethanesulfonic acid (MES) buffer via three rounds of centrifugation at 15,000*g* for 15 min. 1-Ethyl-3-(3-dimethylaminopropyl)carbodiimide (EDC) and *N*-hydroxysuccinimide (NHS) were dissolved at a concentration of 15 and 50 mg/ml in 0.1 M MES (DCN, Carlsbad, CA) at pH 6 before use. MES (0.1 M) at pH 6 was added to the Eu bead solution followed by the addition of 5 μl of EDC and 100 μl of NHS. The reaction mixture was rotated on an orbital shaker with 500 rpm for 30 min at room temperature. The mixture was further purified with three rounds of centrifugation at 15,000*g* for 15 min. After removing the supernatant, 200 μl of 200 mM phosphate buffer and 250 μl of 1 mg/ml Neutravidin (Thermo Fisher Scientific, Waltham, MA) in 10 mM phosphate buffer at pH 7.2 (DCN, Carlsbad, CA) were added. The solution was mixed on an orbital shaker (500 rpm) for 3 hours at room temperature, followed by the addition of 5 μl of 1 M ethanolamine (TCI America, Portland, OR) and continue to shake overnight. The Eu-Neutravidin label was then resuspended in 50 mM tris, 1% casein, 5 mM EDTA, 0.2% Tween 20 of pH 8.0 (DCN, Carlsbad, CA) via three rounds of centrifugation at 15,000*g* for 15 min. This allowed the blocking of Eu-Neutravidin label with casein.

### Spray drying of Eu-Neutravidin on the conjugate pad

Eu-Neutravidin conjugate (60 μl) was added to 1140 μl of trehalose (50 mg/ml) in DI water. The solution was then printed onto the conjugate pad, STD17 (Cytiva, Marlborough, MA), using BioDot Airjet (Irvine, CA). The pads were dried at 40°C for 30 min before placing them into a foil pouch with desiccants (DCN, Carlsbad, CA).

### Lamination of the LFAs

The membrane was first laminated onto the backing card through adhesive (DCN, Carlsbad, CA). This was followed by the lamination of Ahlstrom 270 (Ahlstrom-Munksjö, Helsinki, Finland) on the top of the card as a wicking pad. The conjugate pads were then placed at the other end of the nitrocellulose membrane and secured with adhesives. The backing card was then trimmed to 5-mm strips to allow the strips to be placed into the cassette of the POC reader.

### Detection and quantification of urinary synthetic DNA barcodes with LFA

Tris (25 mM), NaCl (150 mM), 0.5% Pluronic F68 (Thermo Fisher Scientific, Waltham, MA), pH 7.4, was used as the running buffer to perform the measurement. Urine samples (5 μl) were first added to the conjugate pad at a position of ~5 mm away from the nitrocellulose membrane. Ten microliters of the running buffer was then placed 10 mm below the urine samples as a spacer. The strips were then immersed into 125 μl of the running buffer in a 48-well plate. The strips were left to run for 20 min at room temperature to allow the conjugation of DNA barcodes to the Eu-Neutravidin label and also the hybridization at their respective capture probes. The strips were then analyzed with an Axxin AX-2X-S POC reader (Fairfield, VIC, Australia).

### Preparation of dry powder insufflators and intratracheal administration of ABN-laden dry powder aerosols

A dry powder insufflator is designed for rapid, targeted, intratracheal powder administration to mice. It comprises a 1-ml gas-tight syringe (Avanti Lipids), a screen, and a 22-gauge soft catheter (Global Medical Solutions). The fine screens were made from 304 stainless steel wire cloth, 60 × 60 mesh with wire diameter of 0.0114 cm. The screens were cut with tin snips or punched using a die to an outer diameter of 5 mm. To assemble the insufflator, a screen was inserted into the tube of a syringe and placed right next to its blunt needle. The blunt needle of the syringe was then capped by a 22-gauge catheter, which ensures safe intratracheal administration in mice. A predetermined amount of dry powders containing DNA-coded ABNs was carefully loaded into the syringe. The syringe was turned vertical and tapped on the bench to ensure dry powders to cover on the screen. A plunger was slowly inserted into the tube, and 0.5-ml air space was left in the syringe. It is critical not to aerosolize dry powders during plunger insertion. The ABNs with LQ81–DNA barcode 3 (DNA3) were intratracheally aerosolized with the dry powder insufflator to the mice with or without transplanted tumors in the lungs. The urine samples were collected 2 hours after administration. C57BL/6 mice (female, 8 to 10 weeks) were injected with lung epithelial cancer cells isolated from C57BL/6 mice with *Kras* mutation and *p53* inactivation (KP) via tail vein, and tumors were allowed to develop for 2 weeks before dosing the ABNs. DNA3 reporters in the urine were quantified with a LFA immobilizing complementary sequence of DNA3.

### Immunohistochemical staining of ABNs in the lungs

For immunohistochemical visualization of nanosensors following intratracheal administration and nebulization, EZLink NHS-Biotin (Thermo Fisher Scientific) was coupled to amine-terminated PEG_8_40k at a 2:1 molar ratio in dimethyl sulfoxide (DMSO) in the presence of a trace of triethylamine and the reaction was carried out overnight, followed by washes and purification with Millipore Amicon MWCO 10-kDa ultra-0.5 centrifugal filters (Burlington, MA). Pulmonary administration of biotinylated PEG_8_40k nanoscaffolds was performed by intratracheal instillation (50 μl, 20 μM biotin equivalent) or nebulization (0.2 ml, 50 μM biotin equivalent) as described above. Fixation was performed 2 hours after administration by inflating lungs with 1 ml of 4% paraformaldehyde (PFA). Lungs were then excised, fixed in 4% PFA at room temperature overnight, and embedded in paraffin blocks for sectioning. Tissue slices (5 μm) were sectioned and stained for biotin with a streptavidin-HRP (horseradish peroxidase) ABC kit (Vector Laboratories, Burlingame, CA) following a protocol provided by the manufacturer. The stained slides were scanned using the 20× objective of a 3DHistech Pannoramic 250 Flash III whole slide scanner (Budapest, Hungary) and analyzed with QuPath 0.3.0.

### Examination of general histopathological toxicity

A set of 4-plex, DNA-coded ABNs was nebulized to wild-type C57BL/6 mice via an inhalation tower system as mentioned above. The body weights of the mice were monitored for 7 days and normalized to that at the day of ABN nebulization. Lung, liver, heart, spleen, kidney, and brain were carefully excised, fixed in 4% PFA at room temperature overnight, and embedded in paraffin blocks for sectioning. Tissue slices (5 μm) were sectioned and stained for hematoxylin and eosin in the Hope Babette Tang (1983) Histology Facility at the Koch Institute of MIT. All slides were carefully examined by a highly experienced veterinary pathologist (R. Bronson from Harvard Medical School).

### Immunogenicity of DNA-coded nanosensors by ELISPOT

The IFN-γ ELISPOT assay has been used extensively for the screening of immune responses elicited by foreign antigens. A volume of approximately 0.2 ml of blood was sampled into EDTA-coated tubes via retro-orbital bleeding at days 7, 14, and 30 following the nebulization of 4-plex, DNA-coded nanosensor cocktails (375 μM per nanosensor). Red blood cells were lysed with ACK lysing buffers (Thermo Fisher Scientific), and white blood cells were collected by centrifugation at 300*g* for 5 min at 4°C. The cell pellet was resuspended in cold PBS and washed one more time. The resuspended cells were then diluted to 1 million cells/ml with Gibco RPMI 1640 (Thermo Fisher Scientific) supplemented with 10% fetal bovine serum (Thermo Fisher Scientific), and the cells were seeded on a 96-well plate that was precoated with anti-IFN-γ capture antibodies. Pooled 10-μg sterile 4-plex, DNA-coded ABNs were added to the cells and incubated for 24 hours. Secreted IFN-γ was detected with a mouse IFN-γ ELISPOT Pair (BD Biosciences, Franklin Lakes, NJ) following the manufacturer’s protocol. As positive controls, white blood cells isolated from healthy, sex- and age-matched C57BL/6J mice were stimulated with eBioscience cell stimulation cocktail (1×). The eBioscience cell stimulation cocktail (500×) (Thermo Fisher Scientific, Waltham, MA, USA) is a cocktail of 40.5 μM phorbol 12-myristate 13-acetate (PMA) and 670 μM ionomycin in ethanol.
