# Supplementary material for: Inhalable point-of-care urinary diagnostic platform
Source: Sci Adv. 2024 Jan 5;10(1):eadj9591. doi: 10.1126/sciadv.adj9591 (PMC10776015; doi:10.1126/sciadv.adj9591)
Supplement: Supplementary file 1 — Figs. S1 to S11 Tables S1 to S3 [file sciadv.adj9591_sm.pdf]

Science Advances
AAAS

Supplementary Materials for

Inhalable point-of-care urinary diagnostic platform

Qian Zhong *et al.*

Corresponding author: Sangeeta N. Bhatia, sbhatia@mit.edu

Sci. Adv. **10**, eadj9591 (2024)
DOI: 10.1126/sciadv.adj9591

This PDF file includes:

Figs. S1 to S11
Tables S1 to S3

Supplementary Figures

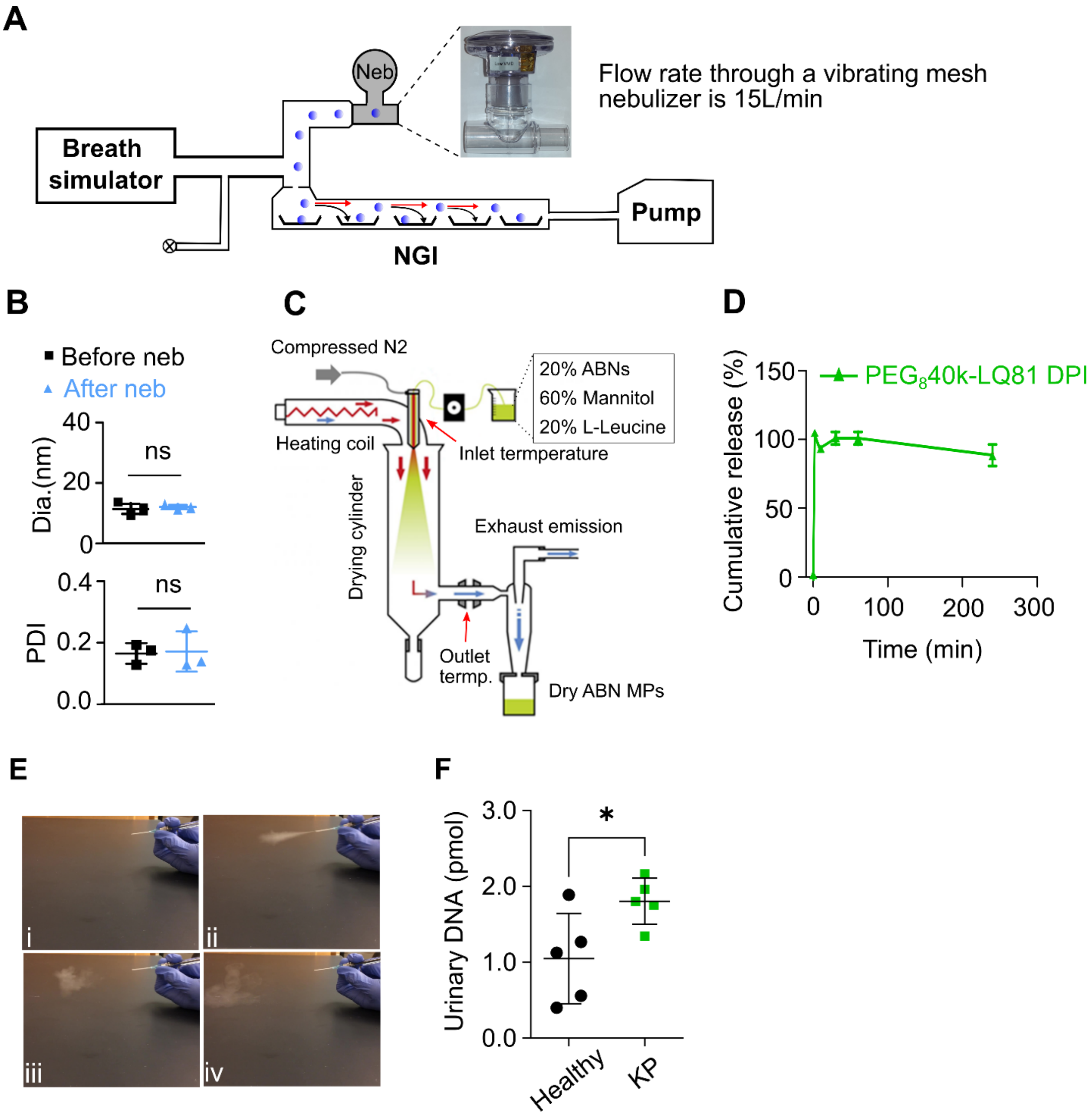

Figure S1. Characterization of aerodynamic performance of ABN aerosols from vibrating mesh nebulizers. (A) A breath simulator that generates and applies inhalation-exhalation profiles that mimics that of a human subject was connected to a next generation impactor (NGI) to improve clinical relevance of the nebulized ABNs in *in-vitro* testing. For instance, delivery dose uniformity and fine particle doses. The tests were carried out under a standardized breathing pattern of 500 mL tidal volume, 1:1 inhalation:exhalation (I:E) ratio, and 15 breaths per minute (BPM) frequency. **(B)** Hydrodynamic diameter (top) and polydispersity (bottom) of the model ABN remained basically unchanged before and after vibrating mesh nebulization (neb). "ns" = not significant. Unpaired Student's t-test was performed between two groups. Abbreviation: Dia: diameter, PDI: polydispersity index. **(C)** Scheme of spray drying process that is utilized to make ABN-laden microparticles. The ABNs (20% by weight) were sprayed into the microparticles using a Buchi Min-290 spray dryer along with pharmaceutical excipients; D-mannitol (60% by weight) and L-leucine (20% by weight). MPs = microparticles. **(D)** ABNs (Cy7-tagged $PEG_8$40k-LQ81) were immediately released from the microparticles when incubating with synthetic lung fluids. One hundred microliters of solution were

added to a 1.5 mL Eppendorf tube and ABNs were isolated by centrifugation. The supernatant was quantified for Cy7 fluorescence. **(E)** The ABN-laden microparticles were aerosolized with a homemade dry powder insufflator (see the details in "Method")**. (F)** Urinary DNA reporters quantified with lateral flow assays immobilizing complementary sequence of DNA barcode 3. Unpaired Student's t test was performed between two groups. *$p<0.05$. The ABNs with LQ81-DNA3 were intratracheally delivered with the dry powder insufflator to the mice with or without transplanted tumors in the lungs. The urine samples were collected 2 hours post administration. KP = female C57BL/6 mice with KP tumors by injecting lung epithelial cancer cells isolated from C57BL/6 mice with Kras mutation and p53 inactivation (KP) via tail vein, and tumors were allowed to developed for 2 weeks before dosing the ABNs.

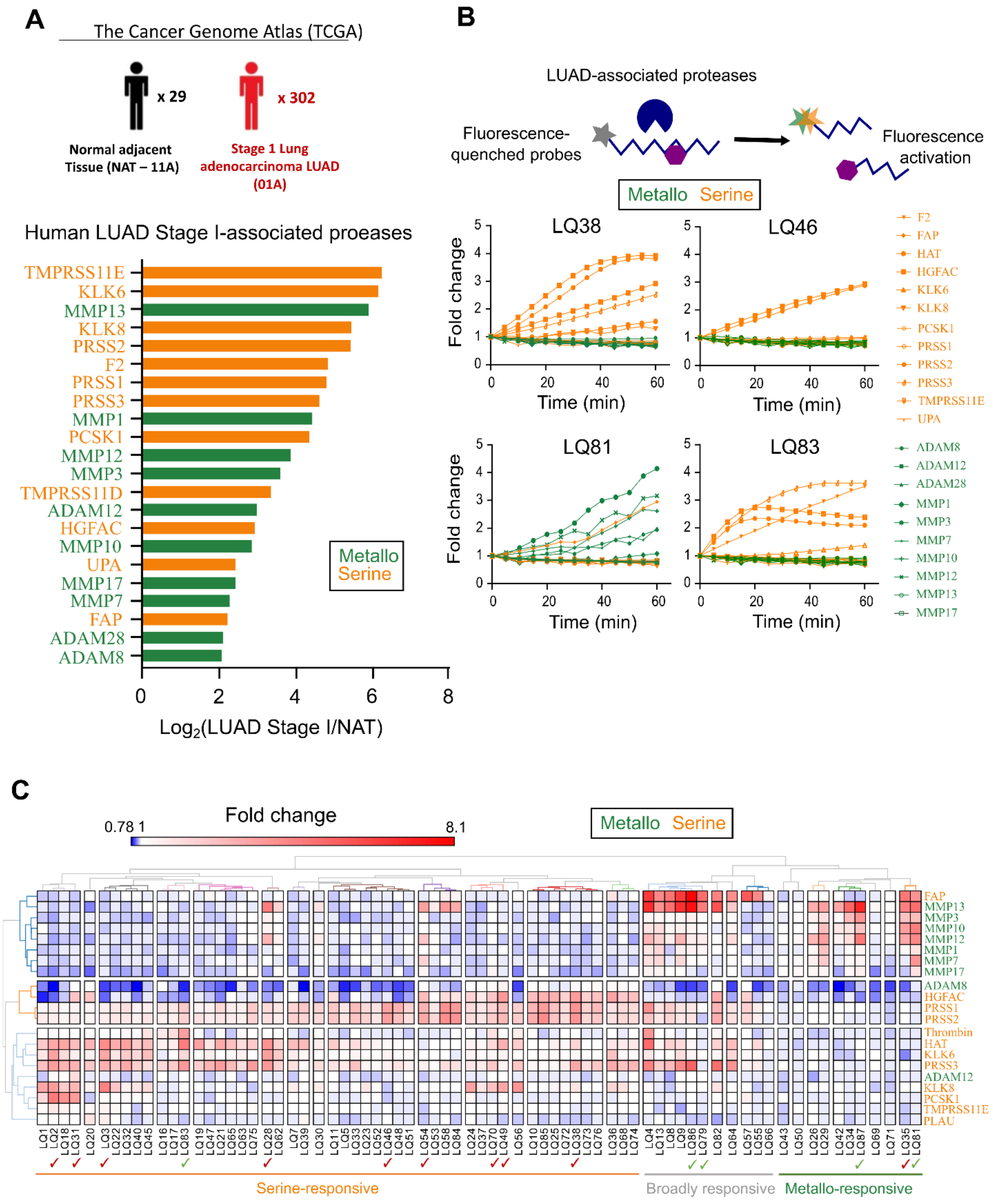

Figure S2. Overexpressed proteases in Stage I are powerful biomarkers to be leveraged for early detection of lung adenocarcinoma (LUAD). (A) Differential gene expression analysis between stage I LUAD and non-tumor adjacent tissue (NAT) using The Cancer Genome Data (TCGA) LUAD data identified top 50 dysregulated proteases. Twenty-two proteases with recombinant counterparts commercially available were plotted and ranked by fold changes. Metalloproteases and serine proteases were color-coded green and orange, respectively. TMPRSS11D/11E: transmembrane serine protease 11D/11E; KLK6/8: kallikrein-6/8; MMP1/3/7/10/12/13/17: matrix metalloproteinases

1/3/7/10/12/13/17; PRSS1/2/3: protease, serine, 1/2/3; F2: thrombin; PCSK1: proprotein convertase 1; ADAM8/12/28: a disintegrin and metalloproteinase 8/12/28; HGFAC: hepatocyte growth factor activator; FAP: Fibroblast activation protein, alpha; UPA (or PLAU): Urokinase-type plasminogen activator. **(B)** A set of 22 proteases dysregulated in Stage I LUAD were screened against of a panel of 15 Förster resonance energy transfer (FRET)–paired protease substrates, and fluorescence activation (green for metallo-proteases and orange for serine proteases) was recorded for 60 min. Fluorescence fold change over 60 min (average of 2 replicates) were z-scored by row and tabulated. Hierarchical clustering was performed to cluster substrates (horizontal) by their protease specificity, Representative kinetic fluorescence curves of LQ38, LQ46, LQ81 and LQ83 against all proteases were shown respectively. The data show that preferential cleavage of LQ81 is by metalloproteases (green) and LQ38, LQ46, LQ83 are by serine proteases (orange). **(C)** Heatmap of z-scored fluorescent fold changes at 50min (average of 2 replicates) showing hierarchical clustering of proteases dysregulated in Stage I LUAD (vertical) by their substrate specificities and of selected FRET-paired synthetic substrates (horizontal) by their protease specificity. Ticks indicate substrates nominated for in vivo evaluation. TMPRSS11D was excluded from the list due to unavailable recombinant counterpart, resulting in 21 proteases.

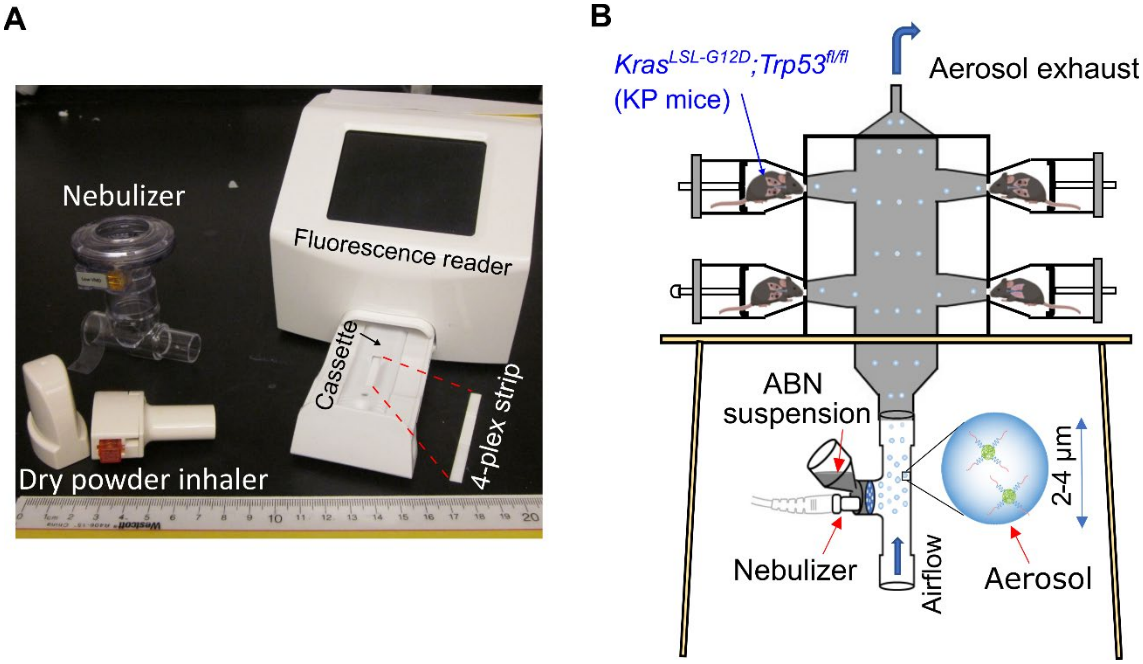

Figure S3. Prototype components for inhalable point-of-care urinary diagnostic platform. (A) Inhalable formulations of the ABNs can be generated via either nebulizers or dry powder inhalers for non-invasive pulmonary delivery. Urine samples are collected and then added on a paper strip to multiplex DNA barcode signals quantitatively with a portable lateral flow assay fluorescent reader. Quantitative readouts of urinary DNA barcodes are used to classify lung cancer groups from healthy cohorts. **(B)** For *in vivo* validation of inhalational delivery of the ABNs, a nose-only exposure inhalation tower was assembled with a vibrating-mesh nebulizer at the bottom and the mice situated in restrainers on the side. The aerosolized ABNs flow into the inhalation tower with medical airs. The mice were only exposed to the ABNs-laden aerosols only via nasal openings. The median volumetric diameter of generated aerosols was in the range of 2-4 μm.

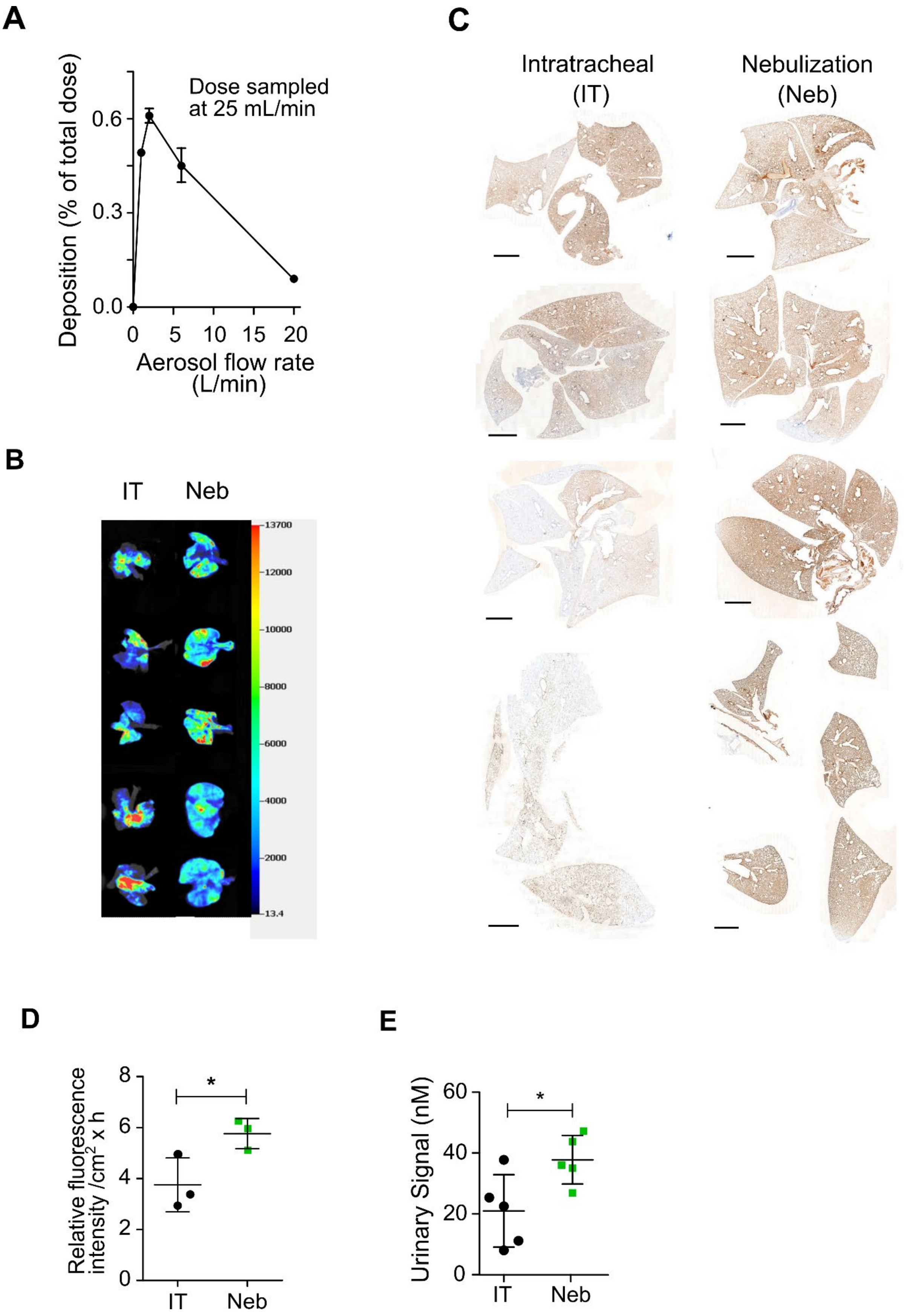
A
Dose sampled at 25 mL/min
Deposition (% of total dose)
0.0
0.3
0.6
0
5
10
15
20
Aerosol flow rate (L/min)
B
IT
Neb
13700
12000
10000
8000
6000
4000
2000
13.4
C
Intratracheal (IT)
Nebulization (Neb)
D
Relative fluorescence intensity /cm^2 x h
0
2
4
6
8
*
IT
Neb
E
Urinary Signal (nM)
0
20
40
60
*
IT
Neb

Figure S4. Local biodistribution of inhaled and intratracheally injected ABNs in lungs. (A) Compressed air flow rate was optimized to maximize ABN aerosols available for animals to inhale. Nebulization outperforming intratracheal injection enables broad and even distribution of ABN in lungs as revealed by **(B)** fluorescence tracers and **(C)** immunohistochemistry staining. A same dose of biotin-labeled ABN was delivered to wild-type C57BL/6 mice and the lungs excised 20 minutes after administration from the intratracheally injected (IT) or nebulization (Neb) cohorts (n=4) were fixed overnight by 4% paraformaldehyde. The fixed lungs were then stained with streptavidin-horseradish peroxidase. The scale bar is 2 mm. **(D)** Plasma concentrations and **(E)** urinary readouts of cleaved reporters in intratracheal and nebulization cohorts. Nebulization groups showed higher area under curve (AUC) of pharmacokinetics and urinary reporter concentrations. Abbreviation: RFU = relative fluorescence units. In (D) and (E), unpaired Student's t test was performed between two groups. *p<0.05.

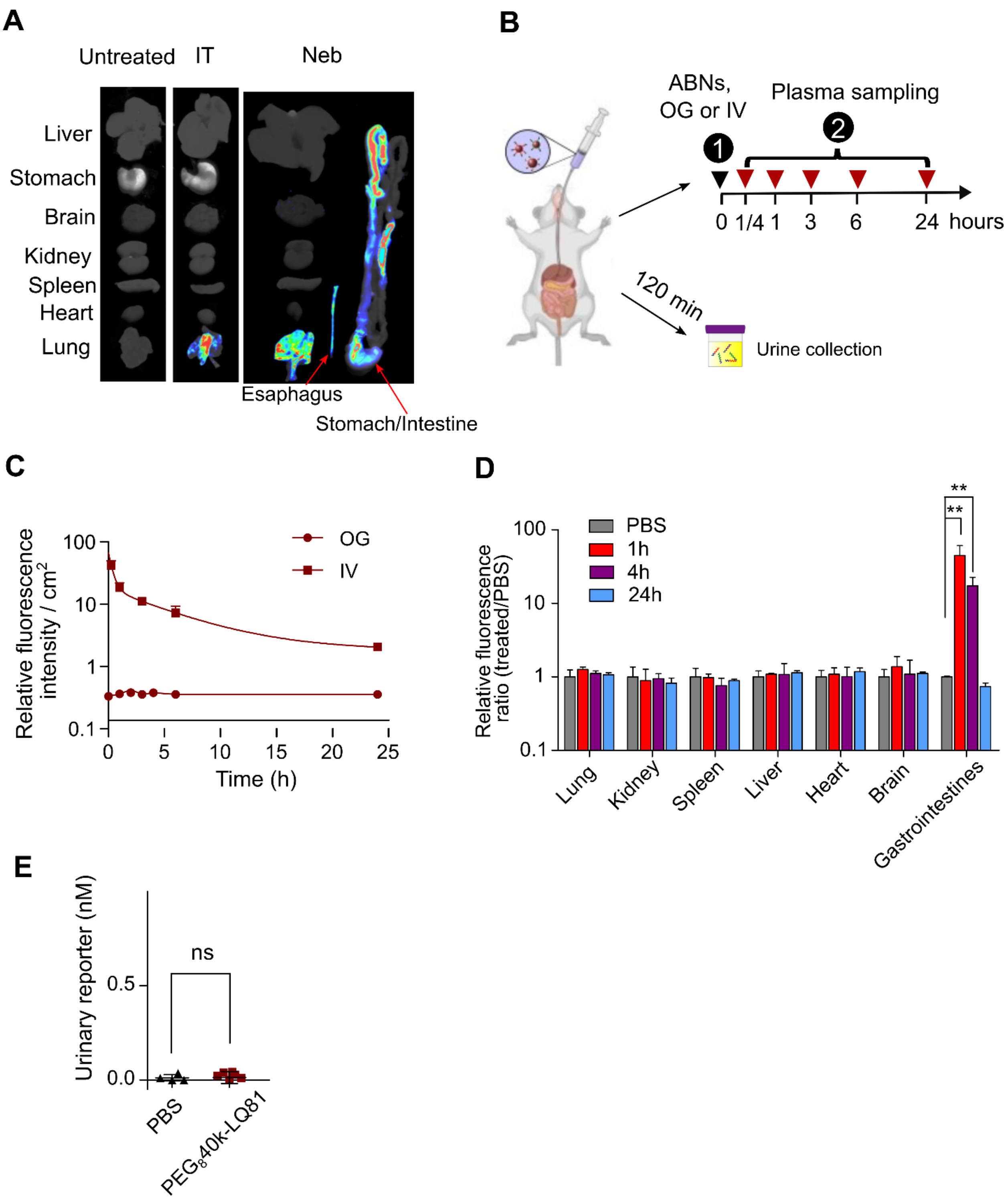

Figure S5. Orally administered ABNs pose no interference with urinary readouts. (A) Representative 1 h biodistribution of intratracheally-instilled (IT) and nebulized (Neb) ABNs that were tagged with Cy7. The ABNs via IT were solely distributed in lungs in a heterogeneous pattern, whereas nebulized ABNs were inhaled into not only the lungs, but digestive system, including esophagus. This poses the question whether those ABNs ending up in gastrointestinal tissues re-enter systemic circulation. **(B)** Schematic illustration of the delivery of Cy7-tagged ABNs

with Glu-fib reporters ($PEG_8$40k-LQ81-Glufib) to animals via oral gavage (OG). The plasma (liquid/cell-free portion of blood) were sampled at various time points (i.e., 2 min, 0.25h, 1 h, 4h, and 24 h post oral administration) to monitor the absorption of ABN into systemic circulation. The urine was collected at 120 min post oral gavage and the fluorescence of Cy7 in urine samples was measured. Intravenously (IV)-injected ABNs was included as a reference. **(C)** Plasma (liquid/cell-free portion of blood) concentration of Cy7 that summed up ABNs and Cy7-tagged reporters liberated from ABNs. Fluorescent signals were not detected in the plasma of the mice treated with orally delivered ABNs, indicating no ABNs or cleaved reporters can re-enter the systemic circulation that ultimately led to urinary concentration. Intravenously injected ABNs (IV) was used as a positive control. **(D)** Systemic biodistribution of ABNs at 1, 4 and 24 h post oral administration. No obvious observation of ABNs in tissues outside the digestive system suggests oral ABNs were excreted without no entry into systemic circulation. Cy7 fluorescence was not detected in the kidneys of mice dosed orally with ABNs, further corroborating undetectable urinary readouts. One-way ANOVA with Dunnett's multiple compassion was performed with respect to the PBS group. *p<0.05, and **p<0.01. **(E)** Urinary readouts of Cy7 fluorescence in the mouse group of oral gavage shows no significant difference in comparison with untreated counterparts, corroborating that orally administered ABNs fail to release fluorescent reporters to the urine. "ns" = not significant. Unpaired Student's t test was performed between two groups.

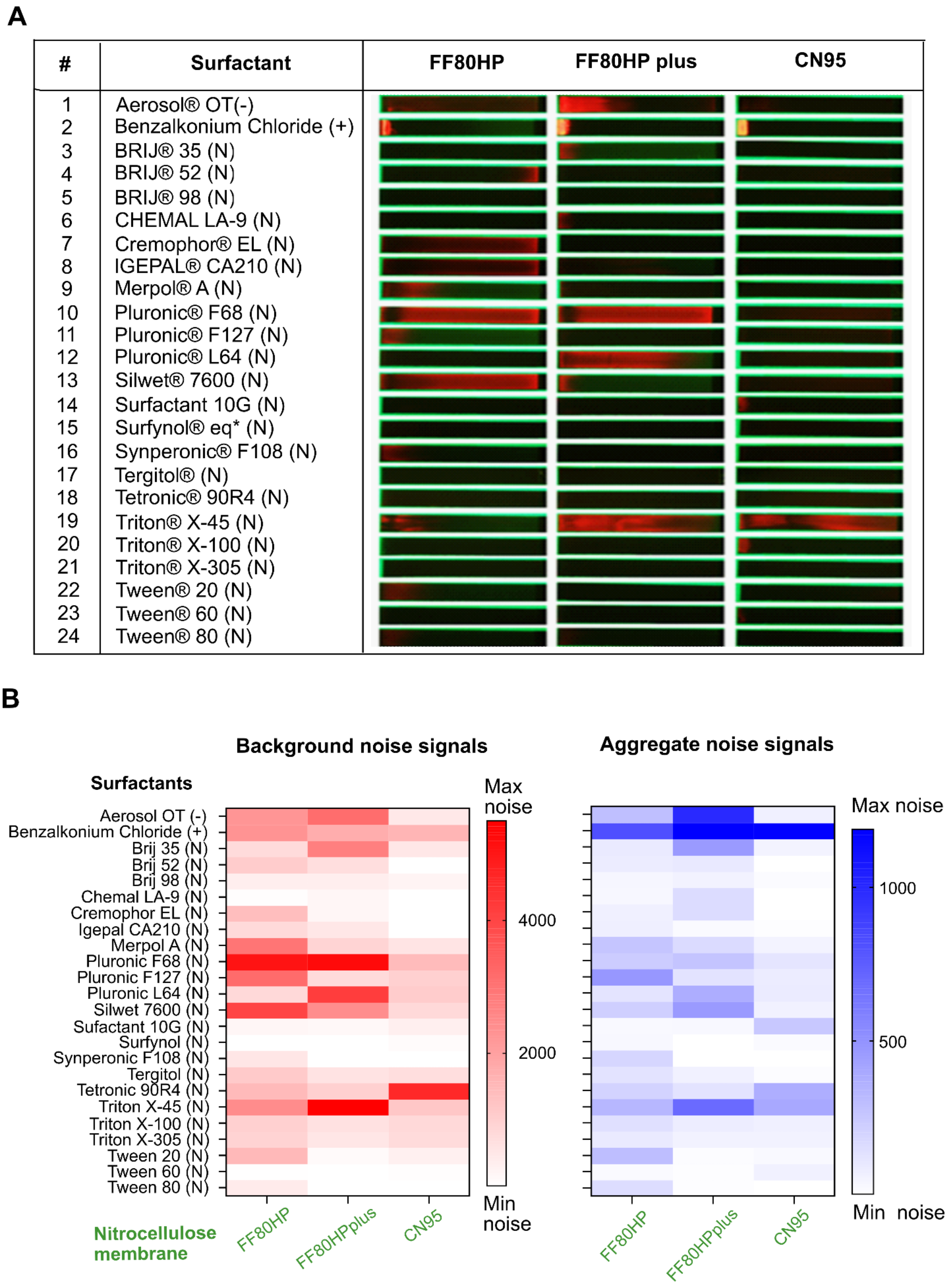
A
#
Surfactant
FF80HP
FF80HP plus
CN95
1 Aerosol® OT(-)
2 Benzalkonium Chloride (+)
3 BRIJ® 35 (N)
4 BRIJ® 52 (N)
5 BRIJ® 98 (N)
6 CHEMAL LA-9 (N)
7 Cremophor® EL (N)
8 IGEPAL® CA210 (N)
9 Merpol® A (N)
10 Pluronic® F68 (N)
11 Pluronic® F127 (N)
12 Pluronic® L64 (N)
13 Silwet® 7600 (N)
14 Surfactant 10G (N)
15 Surfynol® eq* (N)
16 Synperonic® F108 (N)
17 Tergitol® (N)
18 Tetronic® 90R4 (N)
19 Triton® X-45 (N)
20 Triton® X-100 (N)
21 Triton® X-305 (N)
22 Tween® 20 (N)
23 Tween® 60 (N)
24 Tween® 80 (N)
B
Background noise signals
Aggregate noise signals
Surfactants
Aerosol OT (-)
Benzalkonium Chloride (+)
Brij 35 (N)
Brij 52 (N)
Brij 98 (N)
Chemal LA-9 (N)
Cremophor EL (N)
Igepal CA210 (N)
Merpol A (N)
Pluronic F68 (N)
Pluronic F127 (N)
Pluronic L64 (N)
Silwet 7600 (N)
Sufactant 10G (N)
Surfynol (N)
Synperonic F108 (N)
Tergitol (N)
Tetronic 90R4 (N)
Triton X-45 (N)
Triton X-100 (N)
Triton X-305 (N)
Tween 20 (N)
Tween 60 (N)
Tween 80 (N)
Max noise
4000
2000
Min noise
1000
500
Nitrocellulose membrane
FF80HP
FF80HPplus
CN95

Figure S6. Screening of surfactants in running buffer against commercially available membranes. (A) Surf's Up® Surfactant Kit containing up to 24 different surfactants were used for lateral flow assay development. The kit mainly covered non-ionic surfactants but also contained 1 cationic and 1 anionic surfactant. Running buffer prepared using these surfactants were tested against 3 commercially available membranes, namely FF80HP, FF80HP Plus and CN95. Membranes were dipped into the running buffer solution (1% surfactants and 0.5% Eu-conjugates) and the fluorescent signal was recorded. Candidates that 1) did not result in conjugate aggregation at the immersed section of the strip and 2) did not cause high background fluorescence were considered for further development. In other words, strips with minimum fluorescence across the membrane were nominated. * denotes 2,4,7,9-tetramethyl-5-decyne-4,7-diol ethoxylate, which is used as a Surfynol® equivalent. (+) denotes cationic properties, (-) denotes anionic properties, and (N) denotes non-ionic properties. **(B)** Tabulated heat maps indicate the quantification of background noise signals (e.g., Pluronic F68 in **(A)**), implying non-specific binding, and conjugate aggregation signals (e.g., Benzalkonium chloride in **(A)**) at the immersed section of the strip, respectively. To generate the heatmap of background noise signals, Eu fluorescence intensity (illustrated in **Fig. 5C**) profile was first plotted with respect to the distance from the left on the strips using ImageJ. Area under the curve (AUC) of the profile was obtained with GraphPad Prism 5.0. The AUC values were evaluated as background noises (due to non-specific binding). High AUC values indicate high non-specific binding on the strips. Regarding the heatmap of conjugate aggregation, we delineated the first 2.1 mm from the left on the strip as the immersion onset and measured the AUC of Eu intensity profiles in this part.

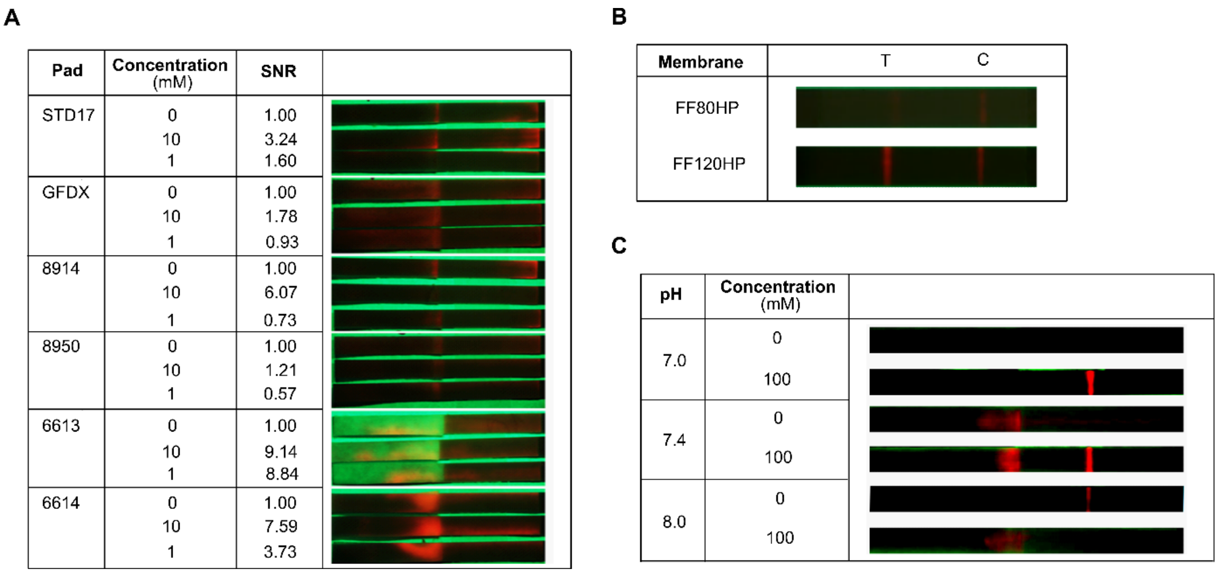

Figure S7. Optimization of lateral flow assay. (A) Screening of conjugate pads for the optimal release of Eu-conjugates. Commercially available conjugate pads containing Eu conjugates were laminated onto FF80HP Plus immobilized with capture probe for barcode 1. Urine containing 0, 1 and 10 nM of the barcode were spotted onto the conjugate pads. Strips were immersed into running buffer of pH 8.6 containing 100 mM Borate, 150 mM NaCl and 10% Tween 60. Criteria to determine optimal performance of the pad included the efficient release of the conjugates (minimal aggregation at the junction between the pads), low background signal and high signal to noise ratio of the barcode. Although the fluorescent signal was not clear visually on the test line, it can be quantified using reader systems. Based on the results, conjugate pad STD 17, 8914 and 8950 were selected for further development. **(B)** Further development of the performance of nitrocellulose membrane. The use of FF120HP Plus with Pluronic F68 showed a more sensitive detection of barcode 2 in comparison to FF80HP previously optimized. Abbreviation: C=control line, T=testing line. **(C)** Optimization of running buffer pH with casein pre-treated Eu-Neutravidin. Casein treatment further resulted in the reduction of background signal on FF120HP Plus. The pH of the running buffer plays a role in the performance of the barcode quantification.

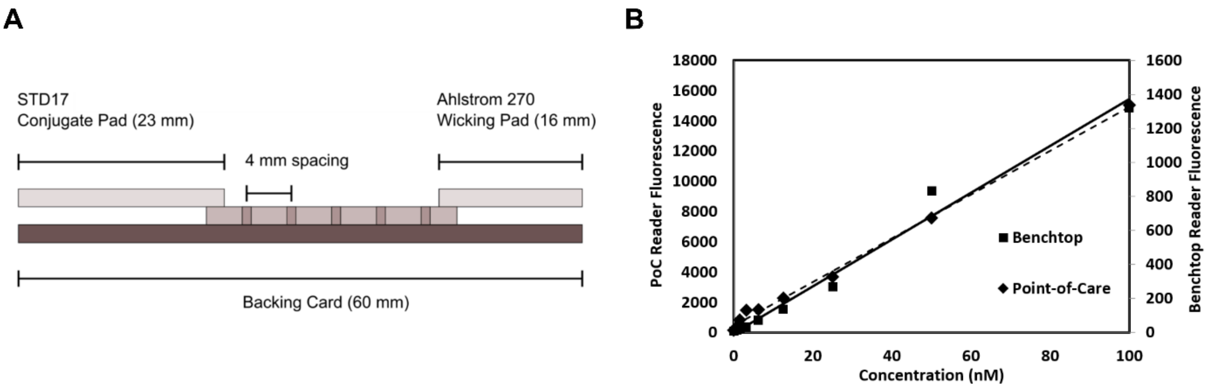

Figure S8. Point-of-care reader compatible lateral flow assay (A) Dimensions of the multiplexed lateral flow assay developed in this study. **(B)** Performance of point-of-care reader vs Qiagen benchtop reader. Both readers show similar performance in the range of interests.

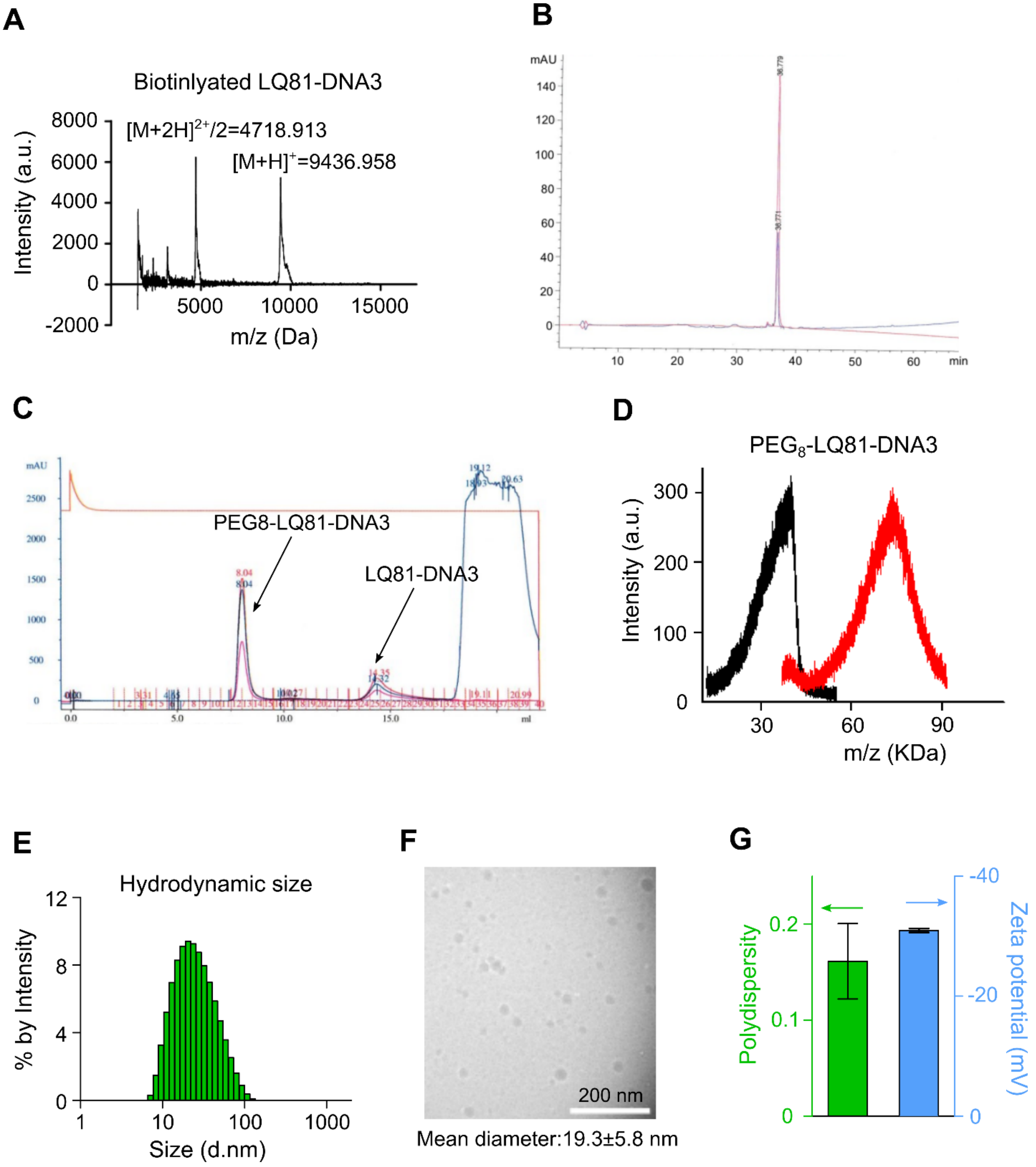

Figure S9. Synthesis and characterization of activity-based nanosensors with synthetic DNA reporters. (A) Mass spectrum of the LQ81-DNA3 conjugate. **(B)** Biotinylated LQ81-DNA3 conjugate was purified with a HPLC equipped with a reverse-phase C18 column to remove unreacted DNA barcodes and peptide LQ81. The eluent composed of acetonitrile and water gradually ramped up the ratio from 10/90 to 90/10 in a period of 30 min. **(C)** $PEG_8$40k-LQ81-DNA3 conjugate was purified with fast protein liquid chromatography (FPLC) with a Superdex 75 10/300 GL column. The conjugate was eluted out at 8.04 min, whereas the unreacted LQ81-DNA3 conjugate was eluted from the column at approximately 14.3 minutes. **(D)** Mass spectrum of the $PEG_8$40k-LQ81-DNA3 conjugate, indicating approximately 4 DNA barcodes were conjugated to each PEG scaffold. Black: 8-arm PEG-maleimide, red:

$PEG_8$40k-LQ81-DNA3 conjugate. **(E)** Dynamic light scattering (DLS) showed hydrodynamic size of mass-coded, 8-arm PEG ABNs is approximately 18 nm. The measurement was performed in 1X PBS. **(F)** Cryo-transmission electron micrograph (Cryo-TEM) of the DNA-coded nanosensors. Geometric size calculated from the Cryo-TEM was 20 nm on average (calculated based on n=50 particles). **(G)** Polydispersity index of 8-arm PEG ABNs measured by DLS was 0.16 and DNA barcodes confer the PEG nanoscaffold negative charges upon conjugation.

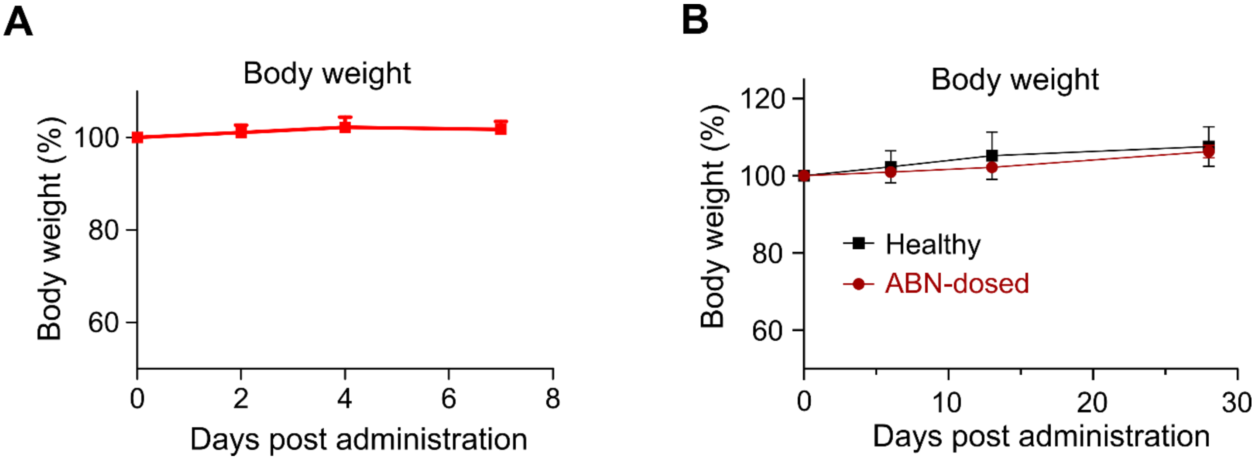

Figure S10. Monitoring change of body weight of mice nebulized with ABNs. A single dose of 4-plex ABNs, 3 folds of the dose used for diagnostic tests, were nebulized to mice (n=5 per cohort) and the body weight were measured at indicated times. **(A)** Body weights of 5 mice remained largely unchanged over 7 days post nebulization of a single dose of 4-plex ABNs. In the cohort, the mice were euthanized and tissues were harvested for hematoxylin/eosin staining (see Fig.7A). **(B)** Body weights of healthy control and ABN-dosed mice over 30 days. These mice (n=5 per cohort) were nebulized DNA-barcoded ABNs for immunogenicity evaluation (see **Fig.7B and C**). Blood samples were collected retro-orbitally at Day 7, 14 and 30 post ABN administration, while the mice were weighed at Day 0, 6, 12 and 28, respectively. We observed no obvious body weight loss over time though the blood samples were drawn 3 times within 30 days, indicating that pulmonary delivery of ABNs imposes no overt toxicity. In summary, constant body weight indicates no acute toxicity was observed associated with inhaled DNA-coded ABNs.

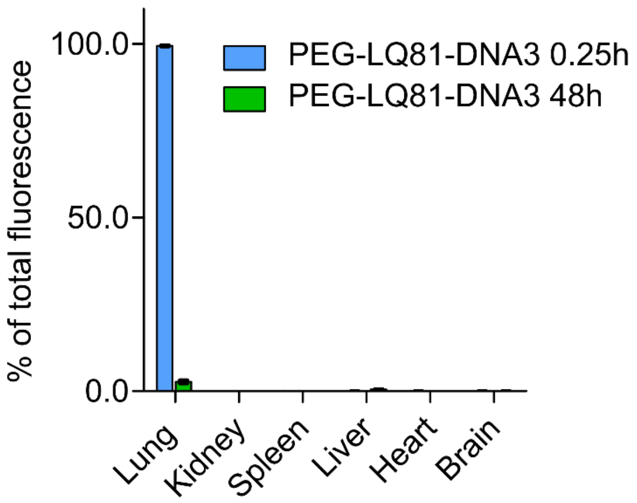

Figure S11. Systemic biodistribution of nebulized DNA-barcoded ABNs. Biodistribution (48h) of DNA-barcoded ABNs delivered via nebulization. In control group (0.25h), the mice were euthanized 0.25 h post ABN nebulization and organs were then harvested to quantify fluorescence from the ABN.

Table S1. Panel of prominent peptide sequences responsive to a library of 21 proteases upregulated in human Stage I LUAD. Uppercase letters denote L-isomers of amino acids, lowercase letters denote D-isomers of amino acids. Abbreviation: GzmB, NE and CatK denote a peptide sequence is cleaved by human granzyme B, neutrophil elastase and cathepsin K, respectively.

Peptide code	Sequences (C terminus= $CONH_2$)
**LQ2**	GGRQARAVGGC
**LQ3**	GGRRARVVGGC
**LQ28**	GGLAQAFRSGC
**LQ31**	GGRQRRALEKGC
**LQ35**	GGPRPFNYLGC
**LQ38**	GGAKIRGQAKGC
**LQ46**	GGSGDRMWggGC
**LQ49**	GGKLRVVGGHPGC
**LQ54**	GGNIPMGLLYNKGC
**LQ70**	GGLGALLRVKRLEC
**LQ79**	GGPQGIWGQGC
**LQ81**	GGPVPLSLVMC
**LQ83**	GGfPRSGGGC
**LQ86**	GGPLGMRGGC
**LQ87**	GGAPFEMSAGC
**P2**	GGPVGLIGC
**P13**	GGP-(Cha)-G-Cys(Me)-HAGC
**GzmB**	GGAIEFDSGC
**NE**	GGAAFAGC
**CatK**	GGHPGGPQC

Table S2. Panel of 20-plexed ABNs. The unique mass-coded glutamic acid-labeled fibroprotein was chemical bonded to each peptide sequence in Table 1 via a photocleavable linker. The entire peptides were then conjugated to 8-arm PEG nanoscaffolds via maleimide-thiol click chemistry. The ABNs were named by adding a prefix 'P' to peptide codes, indicating the PEG nanocore. Uppercase letters denote L-isomers of amino acids, lowercase letters denote D-isomers of amino acids. The ABNs were purified with HPLC and molecular weights were determined with MALDI-TOF.

ABN code	Sequence	Molecular weight (Da)
**PLQ2**	e(+3G)(+1V)ndneeGFFs(+4A)r-ANP-GGRQARAVGGC-(PEG8-40kDa)	65224.0
**PLQ3**	e(+2G)Vndnee(+2G)FFs(+4A)r-(ANP)-GGRRARVVGGC-(PEG8-40kDa)	64035.2
**PLQ28**	eGVndnee(+3G)(+1F)Fs(+4A)r-(ANP)-GGLAQAFRSGC-(PEG8-40kDa)	64640.8
**PLQ31**	e(+2G)(+6V)ndnee(+3G)(+1F)(+1F)s(+1A)r-(ANP)-GGRQRRALEKGC-(PEG8-40kDa)	64769.9
**PLQ35**	e(+3G)(+1V)ndneeG(+10F)FsAr-(ANP)-GGPRPFNYLGC-(PEG8-40kDa)	66570.5
**PLQ38**	e(+2G)Vndnee(+2G)F(+10F)sAr-(ANP)-GGAKIRGQAKGC-(PEG8-40kDa)	63514.2
**PLQ46**	eGVndneeGF(+10F)s(+4A)r-(ANP)-GGSGDRMWggGC-(PEG8-40kDa)	66000.5
**PLQ49**	e(+2G)(+6V)ndneeG(+10F)(+1F)s(+1A)r-(ANP)-GGKLRVVGGHPGC-(PEG8-40kDa)	65018.9
**PLQ54**	eG(+6V)ndneeG(+10F)Fs(+4A)r-(ANP)-GGNIPMGLLYNKGC-(PEG8-40kDa)	67839.9
**PLQ70**	e(+3G)(+1V)ndnee(+2G)(+10F)Fs(+4A)r-(ANP)-GGLGALLRVKRLEC-(PEG8-40kDa)	66952.9
**PLQ79**	e(+2G)Vndnee(+3G)(+10F)(+1F)s(+4A)r-(ANP)-GGPQGIWGQGC-(PEG8-40kDa)	66158.7
**PLQ81**	eGVndneeG(+10F)(+10F)sAr-(ANP)-GGPVPLSLVMC-(PEG8-40kDa)	66457.6
**PLQ83**	eG(+6V)ndnee(+3G)(+1F)Fs(+4A)r-(ANP)-GGfPRSGGGC-(PEG8-40kDa)	60210.3
**PLQ86**	e(+2G)VndneeG(+10F)(+10F)s(+4A)r-(ANP)-GGPLGMRGGC-(PEG8-40kDa)	63497.7
**PLQ87**	e(+2G)(+6V)ndnee(+3G)(+10F)(+1F)s(+4A)r-(ANP)-GGAPFEMSAGC-(PEG8-40kDa)	64116.1
**PP2**	e(+3G)(+1V)ndnee(+2G)(+10F)(+10F)sAr-(ANP)-GGPVGLIGC-(PEG8-40kDa)	60463.1
**PP13**	eGVndnee(+2G)(+10F)(+10F)s(+4A)r-ANP-GGP-(Cha)-G-Cys(Me)-HAGC-(PEG8-40kDa)	60949.4
**PGzmB**	e(+2G)(+6V)ndneeGFFsAr-(ANP)-GGAIEFDSGC-(PEG8-40kDa)	59710.9
**PNE**	eG(+6V)ndneeG(+10F)(+10F)sAr-(ANP)-GGAAFAGC-(PEG8-40kDa)	59565.2
**PCatK**	eG(+6V)ndneeGF(+1F)s(+1A)r-(ANP)-GGHPGGPQC-(PEG8-40kDa)	61364.2

Table S3. DNA sequences of barcodes and capture probes in the study

Barcode	Sequence
**DNA1**	/5BiosG/G*T*T*A*G*T*A*A*G*A*A*C*T*A*T*T*T*G*A*A/3DBCON/
**DNA2**	/5BiosG/T*G*T*C*T*A*T*A*A*A*A*C*A*T*T*A*A*G*A*T/3DBCON/
**DNA3**	/5BiosG/T*C*A*T*A*G*T*T*A*T*C*T*T*A*A*C*A*A*T*C/3DBCON/
**DNA4**	/5BiosG/T*C*A*T*A*G*T*T*A*G*C*G*T*A*A*C*G*A*T*C/3DBCON/
**C1**	/5AmMC6/TTCAAATAGTTCTTACTAAC
**C2**	/5AmMC6/ATCTTAATGTTTTATAGACA
**C3**	/5AmMC6/GATTGTTAAGATAACTATGA
**C4**	/5AmMC6/GATCGTTACGCTAACTATGA

*denotes phosphorothioate bond

/5BiosG/, /3DBCON/ and /5AmMC6/ are the modification code using IDT synthesis service.
